# The fading of reported effectiveness. A meta-analysis of randomised controlled trials

**DOI:** 10.1186/1471-2288-6-25

**Published:** 2006-05-11

**Authors:** Bernhard T Gehr, Christel Weiss, Franz Porzsolt

**Affiliations:** 1 Kreiskrankenhaus Muehldorf, Krankenhausstrasse 1, D – 84453 Muehldorf a. Inn, Germany; 2 Department for Medical Statistics, University Hospital Mannheim, Theodor-Kutzer Ufer, D – 68167 Mannheim, Germany; 3 Clinical Economics, University Hospital Ulm, 89075 Ulm, Germany

## Abstract

**Background:**

The "real" effect size of a medical therapy is constant over time. In contrast, the effect size reported in randomised controlled trials (RCTs) may change over time because the sum of all kinds of bias influencing the reported effectiveness is not necessarily constant. As this would affect the validity of meta-analyses, we tested the hypothesis that the reported effect size decreases over time. Furthermore, we tested three hypotheses that would explain a possible change.

**Methods:**

Because of well established outcome measures, the lipid-lowering drugs Pravastatin and Atorvastatin (serum low-density lipoprotein cholesterol, LDL-C) and the anti-glaucoma drugs Timolol and Latanoprost (intraocular pressure, IOP) were chosen for this investigation. Studies were identified by a standardized MEDLINE search. RCTs investigating the above identified medications administered as monotherapy, and in defined dosages, were included. Publication year, baseline (= pre-treatment value in the treatment group of interest) and post intervention means, number of patients and the assignment to experimental or control group were extracted for each study.

**Results:**

A total of 625 citations were screened; 206 met the inclusion criteria. The reported effect size of Pravastatin (change of reported effect size in five years: -3.22% LDL-C, P < .0001), Timolol (-0.56 mmHg, P < .0001) and Latanoprost (-1.78 mmHg, P = .0074) decreased over time, while there was no significant change for Atorvastatin (+0.31% LDL-C, P = .8618). Multiple regression analysis showed that baseline values were the most important influencing factor; study size or treatment group did not play a significant role.

**Conclusion:**

The effectiveness of medical therapies reported in RCTs decreases over time in three of the four investigated pharmaceuticals, caused mainly by baseline differences. We call this phenomenon "fading of reported effectiveness". Under this condition the validity of a meta-analysis may be impaired. Therefore we propose to observe this phenomenon in future meta-analyses in order to guarantee a maximum of transparency.

## Background

Recently, meta-analyses have become an instrument that is fundamental to the idea of best medical care. Meta-analyses combine the results of a high number of randomised controlled trials (RCTs) to a special topic in order to gain more significant results. Should the reported effect size of RCTs change with time, the result of a meta-analysis would depend on when it was performed. Thus, the validity of a meta-analysis could be impaired.

Nevertheless, an extensive literature search on this topic yielded no results in the medical field, but we identified one relevant study in biology. Jennions and Møller recently examined 44 meta-analyses covering topics like animal behaviour, parasitism and plant growth [1]. They found a small, but highly significant, decline in the strength of reported correlations with publication date (best model: P < .0001; R = -0.133) and with sample size (best model: P < 0.002; R = -0.188). In other words, the investigated meta-analyses would estimate higher intervention effects if they had been performed earlier. The authors attributed the decrease to Publication Bias (under-reporting of studies with small sample sizes and little effect) and Time of Publication Bias (studies that report large effect sizes are published sooner than other studies). Unfortunately, they did not investigate if sample size increased with publication year and they did not describe the time lag between study completion and publication date for the individual studies which would have been necessary to verifying their hypotheses. Moreover, as it remains unclear whether results from the field of biology apply as well to medical research, this paper reports a meta-analysis of clinical drug trials to examine the reported effectiveness of medical treatments over time.

The objective of our longitudinal meta-analysis was to determine if the effect size of medical therapies, as reported in RCTs, changes with time. Further we aimed to identify reasons for any possible change. The unit of analysis was the individual study and not the individual trial participant.

But why should the effect size of a medical intervention change with time? Our hypothesis is based on the assumption that we have to distinguish between the "real" effectiveness of a medical therapy and the effectiveness reported in RCTs, and that the latter may change with time.

(1) The true biological effect size of a medical therapy is constant over time, meaning it should be possible to obtain similar results when a trial is repeated at a later date under identical conditions.

(2) The sum of all kinds of bias influencing the reported effect size of a medical therapy is not necessarily constant over time. In the course of time social, political and economic circumstances for medical research and its publication change. Therefore, it can be assumed that the impact of the various potential sources of bias changes with time in a highly dynamic way. In consequence, the effect size of a medical therapy, as reported in RCTs, may change over time.

As we presumed that, if there was any change at all, the reported effectiveness of medical therapies was more likely to decrease than to increase, we tested three hypotheses that might explain a decrease in the reported effect size:

### (a) Decreasing Publication Bias

The problem of Publication Bias is well known in the medical literature [2-5]. Studies with positive outcomes and significant results are more likely to get published, leading to an under-representation of studies with negative or non-significant results in meta-analyses. Since the level of significance rises with increasing study size, the problem of Publication Bias more likely applies to studies with smaller sample sizes. New medical therapies are first tried in small selected populations, followed by bigger trials, with the aim of validating the benefits in larger populations. Increasing study size should lead to a decrease in Publication Bias, and to lower reported effect sizes over time.

### (b) Spectrum bias (a particular form of selection bias)

New medical interventions tend to be studied in severely ill patients where significant benefits can be expected. After a therapy is established, physicians tend to broaden its use and prescribe it to a wide range of patients, including a high number of less sick patients. In addition, specific treatment goals have been developed in recent years for several diseases such as hypertension, diabetes mellitus and glaucoma; patients who might not have been treated a decade ago, today receive therapy. In less sick patients, less improvement of the study parameter can be expected. Over time the effectiveness of the therapy seems to diminish.

### (c) "Shift of treatment group" bias

Although the studies were conducted as RCTs, expectations of patients, physicians and study authors may play a role, favouring the therapy used in the experimental treatment group. Over time, medical therapies originally considered as innovative therapies become established, and, in later studies, are no longer innovative and therefore implemented as control therapies. This may lead to a decrease in the reported effect size over time.

To answer our questions we examined data from a large number of RCTs dealing with the effectiveness of four different pharmaceuticals. The primary outcome measure was the reported effect size; secondary outcome measures were the publication year, the study size, the mean pre-intervention level of the investigated parameter and the treatment assignment to experimental or control group.

## Methods

### Selection of pharmaceuticals

The pharmaceuticals to be investigated in this experiment had to comply with the following requirements: (1) Their effectiveness was measurable in terms of commonly accepted quantitative parameters that are reported in most studies; (2) the pharmaceuticals were administered as a monotherapy, and in a fixed dosage in order to obtain a high number of studies with comparable results; (3) the therapies were of clinical importance and of general interest. For our investigation, we arbitrarily chose the lipid-lowering drugs Pravastatin and Atorvastatin (route of administration: oral; outcome measure: change of serum low-density lipoprotein cholesterol) and the anti-glaucoma drugs Timolol and Latanoprost (eye drops; change of intraocular pressure).

### Data sources

A standardized literature search was performed with emphasis on transparency and repeatability rather than on completeness. Our MEDLINE search strategy included the following text strings [6]: "Pravastatin LDL", "Atorvastatin LDL", "Timolol glaucoma", and "Latanoprost glaucoma". The literature search was performed for the time up to and including December 2001. The MEDLINE search was limited to studies on human subjects and to items with abstracts only. A filter for randomised controlled trials was used. Non-English studies were included.

### Study selection

**Figure 1:**
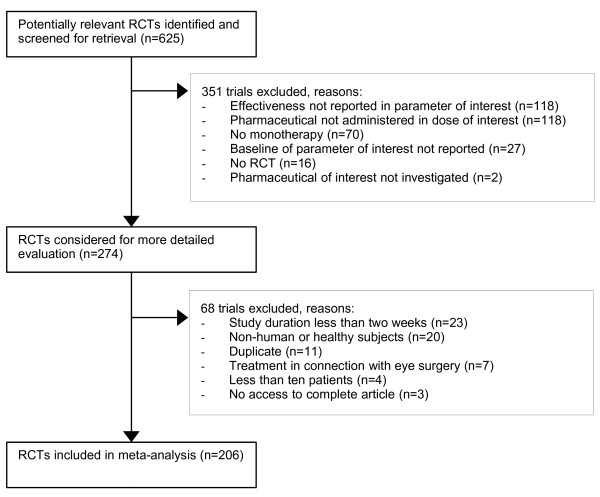
Flow diagram. Abbreviations: RCT, randomized controlled trial; n, number of trials. * Parameter of interest: Reported effectiveness of the pharmaceutical, measured as change of intraocular pressure (Timolol, Latanoprost) or change in low-density lipoprotein cholesterol (Pravastatin, Atorvastatin). ** Dose of interest: In terms of comparability studies that did not use the pharmaceutical in the most common dosage were excluded, as well as studies that increased the individual dosage until a certain outcome was reached.

A study was included if it met the following criteria: (1) baseline value and post-intervention value of the parameter of interest were reported, i. e. low-density lipoprotein cholesterol for Pravastatin and Atorvastatin, intraocular pressure for Timolol and Latanoprost; (2) the pharmaceutical was administered as monotherapy and after a wash-out period; (3) the pharmaceutical was administered in the most commonly used dosage, i. e. Pravastatin 40 mg once daily, Atorvastatin 10 mg once daily, Timolol 0.5 % twice daily, and Latanoprost 0.005 % once daily; (4) the study was conducted as a randomised controlled trial. One investigator (BTG) reviewed 625 citations and selected appropriate studies. 274 studies were considered for more detailed evaluation. Eventually, 206 studies were deemed appropriate for inclusion (Figure 1).

### Data extraction

One of the authors (BTG) extracted the following data for each study: publication year, study size (number of evaluated patients), pre- and post-intervention mean values of low-density lipoprotein cholesterol/intraocular pressure (baseline = pre-treatment value in the treatment group of interest), and assignment to experimental or control group.

Where necessary, means were approximated from figures in the manuscripts or calculated from individual patient data. In each study, the effect size for the intervention was calculated by the difference between the means of the treatment group of interest before and after intervention. In some studies, more than one post-intervention mean was reported; e. g., for different follow-up visits or for different hours of the day. In these cases, the arithmetic mean of the given means was calculated instead of choosing one of the given means arbitrarily. Unfortunately, standard deviations of effect sizes could not generally be reported as they were not included in all original papers. For the same reason it was not possible to perform a weighted analysis.

A study was given the designation "control group", if the pharmaceutical of interest was compared with at least one newer pharmaceutical. The designation "experimental group" was chosen if the pharmaceutical of interest was compared with older pharmaceuticals, or if no other pharmaceuticals were involved in the study (e. g. placebo-controlled studies or studies comparing the effectiveness of different dosages of the same pharmaceutical).

We realized that it was difficult to find independent studies published over a period of 10 years which investigated exactly the same treatment in "exactly" the same patients. Unfortunately, it was not possible to find studies that used – in addition to the above criteria – the same comparative treatments. We found many consecutive studies but the comparative treatments used in these studies varied from trial to trial. As a consequence the other treatments that varied from study to study were not included in our analysis.

### Statistical analysis

Statistical analysis was performed with SAS software (SAS release 8.02, SAS Institute Incorporated, Cary, USA). For each of the variables (publication year, reported effect size, baseline and study size) arithmetic means and standard deviations were calculated. The reported effect size was measured in the most commonly reported dimension (Pravastatin and Atorvastatin: change of low-density lipoprotein cholesterol in percentages; Timolol and Latanoprost: change of intraocular pressure in mmHg). As the variable treatment group is dichotomous with the two possibilities experimental and control group, exact frequencies are reported. In addition, we calculated the arithmetic mean of the publication year of those studies coded as "control" versus those coded as "experimental".

We examined the association between year and effect size and the association between year and other study characteristics. The primary outcome variable (reported effect size) and the secondary outcome variables (baseline, study size and treatment group) were regressed against the publication year. For the variable treatment group, point biserial correlation was used (control group = 0, experimental group = 1). For every correlation the equation of the regression line and the limits of its 95 percent confidence intervals (CI) were calculated (a positive correlation would mean increasing effect sizes with time, a negative correlation decreasing effect sizes with time). With that, the mean change of every variable during an interval of five years (± 95 % CI) was calculated (a positive sign on the mean change would mean an increase, a negative sign a decrease with time).

Pearson correlation coefficients and P-values were calculated for all variables that may influence the effect size. We used a standard approach for statistical significance (α = 0.05). The funnel plot technique was used to detect publication bias [7-9]: Diagrams of the relation between study size and reported effect size were drawn and visually checked for asymmetry.

Furthermore we quantified the impact of the different variables on the reported effect size. For this, we performed a multiple regression analysis with reported effect size as the outcome variable and publication year, baseline, study size, and treatment group as possible predictors. Up to two predictors were entered into the model.

We investigated if measuring the primary outcome variable in absolute or relative dimensions changes the significance levels of the results. Bivariate qualitative analysis and multiple regression analysis were performed with reported effect size measured in absolute terms (Pravastatin and Atorvastatin: low-density lipoprotein cholesterol change in mg/dl, Timolol and Latanoprost: intraocular pressure change in mmHg) and in relative terms (change of outcome variable in percentages).

We could not investigate the change of reported effectiveness in the control therapies of the pharmaceutical of interest, because the control therapies were different in almost any of the studies. The chance to find a study comparing exactly the same control and experimental group several years later is rather low.

## Results

### Included trials and trial characteristics

A total of 206 studies were included in the final analysis (Figure 1). 64 of the included studies investigate Pravastatin, 35 Atorvastatin, 75 Timolol, and 32 Latanoprost. For a list of the individual trials [see Additional file 1], for the extracted raw data [see Additional file 2].

Table 1 shows the mean (± standard deviation, SD) year of publication, reported effect size, baseline value, and study size for each of the four investigated medical therapies. For the dichotomous variable treatment group (experimental/control group) exact frequencies are given, and in addition the mean year of publication in dependence of the treatment group. Pravastatin lowered the low-density lipoprotein cholesterol (LDL-C) on average by 29.50 percent (± 4.16), Atorvastatin by 36.07 percent (± 3.70); Timolol lowered the intraocular pressure (IOP) on average by 6.55 mmHg (± 1.56), Latanoprost by 6.83 mmHg (± 1.53).

**Table 1:** Trial characteristics

	**Pravastatin (n = 64)**				**Atorvastatin (n = 35)**			
	Mean	± SD	Min.	Max.	Mean	± SD	Min.	Max.
Year of publication	1995.28	± 3.46	1990	2001	1999.40	± 1.82	1996	2001
Reported effect size*	-29.50	± 4.16	-19.0	-39.0	-36.07	± 3.70	-28.4	-44.2
Baseline^†^	205.62	± 47.78	134.4	344.0	198.05	± 35.05	143.0	340.3
Study size^‡^	595.77	± 1511.59	10	9014	328.71	± 686.64	22	3916

Treatment group^§^	EG: 56 (87%); CG: 8 (13%)				EG: 31 (89%); CG: 4 (11%)			

	**Timolol (n = 75)**				**Latanoprost (n = 32)**			
	Mean	± SD	Min.	Max.	Mean	± SD	Min.	Max.

Year of publication	1992.68	± 6.99	1978	2001	1999.06	± 2.00	1995	2001
Reported effect size	-6.55	± 1.56	-3.65	-11.3	-6.83	± 1.53	-3.5	-9.8
Baseline	25.94	± 2.49	20.8	38.7	24.07	± 2.13	19.3	28.2
Study size	197.96	± 249.59	12	1198	152.81	± 195.57	20	829

Treatment group	EG: 12 (16%); CG: 63 (84%)				EG: 29 (91%); CG: 3 (9%)			

Abbreviations: n, number of trials that met selection criteria; SD, standard deviation; EG, experimental group; CG, control group.

* Unit of measurement for reported effect size: Change of intraocular pressure measured in mmHg (Timolol, Latanoprost), change in low-density lipoprotein cholesterol measured in % (Pravastatin, Atorvastatin).

^† ^Unit of measurement for baseline: Intraocular pressure measured in mmHg (Timolol, Latanoprost), low-density lipoprotein cholesterol measured in mg/dl (Pravastatin, Atorvastatin). To convert low-density lipoprotein cholesterol from milligrams per deciliter to millimoles per liter, multiply milligrams per deciliter by 0.0259.

^‡ ^Unit of measurement for study size: Number of patients included in final analysis.

^§ ^As the variable "treatment group" is either "experimental group" or "control group", exact frequencies and percentages are given.

### Effect of time on investigated variables

**Figure 2:**
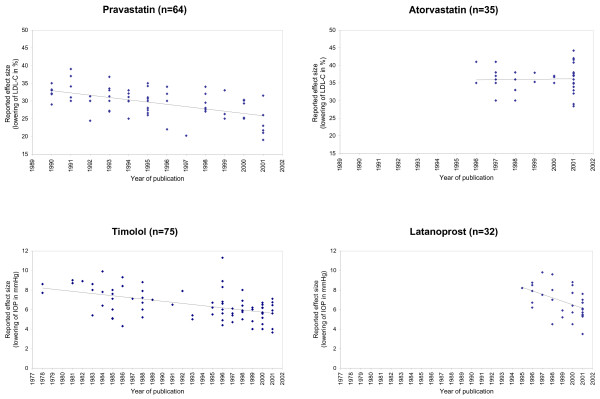
Regression analysis of the relation between year of publication and reported effectiveness of Pravastatin (y = 90.818 – 0.643x; P < 0.0001), Atorvastatin (y = 29.900 + 0.062x; P = 0.8618, not significant), Timolol (y = 16.983 – 0.113x; P < 0.0001), and Latanoprost (y = 42.069 – 0.356x; P = 0.0074). Abbreviations: IOP, intraocular pressure; LDL-C, low-density lipoprotein cholesterol; x, reported effectiveness (for Timolol and Latanoprost change of IOP measured in mmHg, for Pravastatin and Atorvastatin change of LDL-C measured in %); y, year of publication minus 1900.

Over time, the reported effect size decreased significantly for three of the four investigated pharmaceuticals (Figure 2; Table 2 and 3). Pravastatin on average was reported to lower the patient's LDL-cholesterol by 3.22 percent less every five years (95% confidence interval: ± 1.28; P < 0.0001), meaning e. g. by 29.74 percent in 1995 and by 26.52 percent in 2000. Timolol was reported to reduce the intraocular pressure by 0.56 mmHg less every five years (± 0.22; P < 0.0001), Latanoprost by 1.78 mmHg less every five years (± 1.26; P = 0.0074). For Atorvastatin, the reported effect size did not change significantly over time (P = 0.8618).

**Table 2:** Bivariate quantitative analyses of all investigated parameters in dependence of year of publication. All given data are calculated from the equations of the regression lines. For P-values see table 3

		**Pravastatin**	**Atorvastatin**	**Timolol**	**Latanoprost**
Reported effect size*	Change in 5 years	-3.22	+0.31	-0.56	-1.78
	95% CI limits	(-4.50/-1.93)	(-3.29/+3.91)	(-0.79/-0.34)	(-3.04/-0.51)
Baseline^†^	Change in 5 years	-41.80	-14.63	-0.70	-1.82
	95% CI limits	(-55.74/-27.86)	(-48.38/+19.11)	(-1.08/-0.32)	(-3.69/+0.05)
Study size^‡^	Change in 5 years	+533.54	+233.63	+80.55	-16.94
	95% CI limits	(-3.94/+1071.01)	(-429.97/+897.23)	(+43.38/+117.71)	(-199.23/+165.35)
Treatment group^§^	Change in 5 years	-0.20	-0.28	-0.12	-0.23
	95% CI limits	(-0.31/-0.08)	(-0.58/+0.01)	(-0.17/-0.06)	(-0.50/+0.03)

Abbreviations: CI, confidence interval; LDL-C, low-density lipoprotein cholesterol.

* Unit of measurement for reported effect size: Change of intraocular pressure measured in mmHg (Timolol, Latanoprost), change in low-density lipoprotein cholesterol measured in % (Pravastatin, Atorvastatin).

^† ^Unit of measurement for baseline: Intraocular pressure measured in mmHg (Timolol, Latanoprost), low-density lipoprotein cholesterol measured in mg/dl (Pravastatin, Atorvastatin). To convert low-density lipoprotein cholesterol from milligrams per deciliter to millimoles per liter, multiply milligrams per deciliter by 0.0259.

^‡ ^Unit of measurement for study size: Number of patients included in final analysis.

^§ ^The parameter treatment group has two possibilities: control group = 0, experimental group = 1. Point biserial correlation was used to obtain the equation of the regression line and to calculate the given data.

**Table 3:** Bivariate qualitative analyses of all investigated parameters. Significant correlations (P < 0.05) are highlighted by bold digits

	Reported effect size*			Baseline			Study size			Treatment group^†^		
	Ph	R^‡^	P-value	Ph	R	P-value	Ph	R	P-value	Ph	R	P-value
Year of Publication	**P:**	**-0.5360**	**<.0001**	**P:**	**-0.6056**	**<.0001**	P:	0.2444	0.0517	**P:**	**-0.4092**	**0.0008**
	A:	0.0305	0.8618	A:	-0.1518	0.3840	A:	0.1237	0.4789	A:	-0.3207	0.0603
	**T:**	**-0.5043**	**<.0001**	**T:**	**-0.3955**	**0.0004**	**T:**	**0.4512**	**<.0001**	**T:**	**-0.4513**	**<.0001**
	**L:**	**-0.4642**	**0.0074**	L:	-0.3413	0.0559	L:	-0.0346	0.8508	L:	-0.3167	0.0774

Reported effect size	1.00			**P:**	**0.2591**	**0.0387**	P:	-0.1899	0.1327	P:	0.1598	0.2073
				A:	-0.1454	0.4045	A:	0.0381	0.8281	**A:**	**-0.4339**	**0.0092**
				**T:**	**0.6949**	**<.0001**	T:	-0.1364	0.2432	T:	0.1565	0.1801
				**L:**	**0.8745**	**<.0001**	L:	0.2206	0.2251	L:	0.0289	0.8753

Baseline				1.00			**P:**	**-0.2897**	**0.0202**	P:	0.0872	0.4933
							A:	-0.0868	0.6201	A:	0.0848	0.6282
							T:	-0.1923	0.0983	T:	0.0686	0.5587
							L:	0.2673	0.1392	L:	-0.1069	0.5604

Study size							1.00			P:	0.0566	0.6568
										A:	0.1106	0.5269
										**T:**	**-0.2723**	**0.0181**
										**L:**	**-0.3557**	**0.0457**

Abbreviations: Ph, pharmaceutical; T, Timolol; L, Latanoprost; P, Pravastatin; A, Atorvastatin.

* Reported effect size for Pravastatin and Atorvastatin is measured in relative terms (%), for Timolol and Latanoprost in absolute terms (mmHg).

^† ^As the parameter treatment group only has the possibilities control group (= 0) or experimental group (= 1), point biserial correlation was used.

^‡ ^R: Pearson correlation coefficient.

Most of the other investigated variables changed over time as well (Table 2 and 3). The baseline values of the variable of interest decreased over time for all investigated pharmaceuticals. This relation was significant for Pravastatin (-41.80 mg/dl LDL-C every five years; P < 0.0001) and Timolol (-0.70 mmHg IOP every five years; P = 0.0004). Study size increased over time for three of the four pharmaceuticals; this relation was significant only for Timolol (+80.55 patients every five years; P < 0.0001). The variable treatment group changed over time from experimental group towards control group for all investigated pharmaceuticals. This relation was significant for Pravastatin (-0.20 every five years, meaning that in all Pravastatin studies, every five years 20 percent less used Pravastatin in the experimental treatment arm and 20 percent more in the control arm; P = .0008) and for Timolol (-0.12 every five years ; P < .0001).

### Other bivariate analyses

For Pravastatin, Timolol and Latanoprost the reported effect size correlated significantly with the baseline values of the variable of interest (Table 3). The reported effect size was related to treatment group for Atorvastatin (P = 0.0092). There was no significant correlation between study size and reported effect size (in the most commonly reported dimension) for any of the investigated pharmaceuticals.

### Measuring effect size in relative or absolute dimensions

Some results of the lipid lowering drugs were altered when the reported effect size was measured in absolute and not in relative terms. When measuring the change of LDL-C in mg/dl and not in percentages, (1) the relation between reported effect size and baseline value *was *significant for Atorvastatin (P < .0001 vs. P = 0.4045), (2)the relation between reported effect size and treatment group *was not *any more significant for Atorvastatin (P = 0.3731 vs. P = 0.0092), and (3) the relation between reported effect size and study size *was *significant for Pravastatin (P = 0.0139 vs. P = 0.1327). For Timolol and Latanoprost the results were not altered if the reported effect size was measured in relative terms and not in absolute terms.

### Multiple regression analysis

The results of the multiple regression analysis differed if the effect size was measured in absolute or in relative terms. If measured in absolute terms the baseline was the most reliable predictor and alone explained 80.37 percent of the variability of the reported effect size of Pravastatin (R^2^; Table 4), 69.59 percent of Atorvastatin, 48.29 of Timolol and 76.47 percent of Latanoprost. If the variables "publication year" or "treatment group" were entered an additional 3.11–6.24 percent of the variability were explained by the model. The variable "study size" added not more than 0.23 percent.

**Table 4:** Multiple regression analysis to explain the variability of the parameter "reported effect size". Shown are the top four models taking into account one or two variables with the effect size measured in absolute or in relative dimensions

	Ranking of model	Effect size measured in absolute terms		Effect size measured in relative terms	
		Variables in model	R^2^*	Variables in model	R^2^
**Pravastatin**	1	B, Y	0.8477	Y, B	0.2941
	2	B, T	0.8077	Y, T	0.2916
	3	B, n	0.8060	Y, n	0.2910
	4	B	0.8037	Y	0.2873

**Atorvastatin**	1	B, T	0.7473	T, Y	0.2014
	2	B, Y	0.6961	T, B	0.2001
	3	B, n	0.6960	T, n	0.1957
	4	B	0.6959	T	0.1882

**Timolol**	1	B, Y	0.5453	Y, B	0.2351
	2	B, T	0.4948	Y, n	0.2206
	3	B, n	0.4829	Y, T	0.1979
	4	B	0.4829	Y	0.1958

**Latanoprost**	1	B, Y	0.7958	B, Y	0.5636
	2	B, T	0.7798	B, T	0.5306
	3	B, n	0.7649	B, n	0.5030
	4	B	0.7647	B	0.5030

Abbreviations: Y, year of publication; B, baseline of parameter of interest; T, treatment group; n, study size.

* R^2^: Determination coefficient.

If measuring the effect size in relative terms the results of the multiple regression analysis were less homogenous, but overall the year of publication was the most important predictor for the reported effect size. For Pravastatin and Timolol the variable "publication year" alone explained 28.73/19.58 percent of the effect size variability (R^2^; Table 4). For Pravastatin other variables did not add more than 0.68 percent when entered in the model, for Timolol "baseline value" added 3.93 and "study size" 2.48 percent. For the reported effect size of Atorvastatin, "treatment group" was the most important predictor (R^2 ^= .1882); of Latanoprost, the "baseline value" (R^2 ^= .5030).

### Evaluation of potential bias

**Figure 3:**
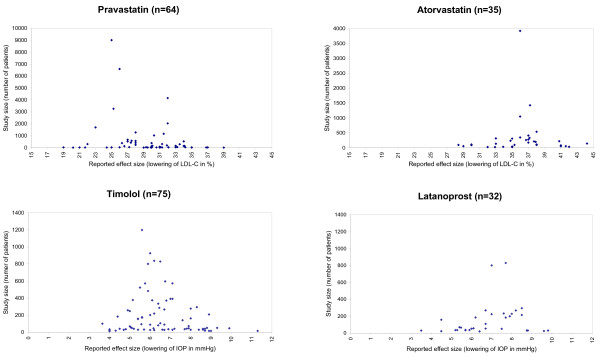
Funnel plots; shown is the relation between study size and reported effectiveness. None of the relations are statistically significant (Pravastatin P = 0.1327; Atorvastatin P = 0.8281; Timolol P = 0.2432; Latanoprost: P = 0.2251). Abbreviations: n, number of trials; IOP, intraocular pressure; LDL-C, low-density lipoprotein cholesterol.

The funnel plot technique was used to evaluate Publication Bias. The study size was plotted against the reported effect size of the study (Figure 3). The plots of Atorvastatin and Latanoprost did not show relevant asymmetry, an indication that significant Publication Bias was unlikely. The plots of Pravastatin and Timolol showed slight asymmetry. For example the Timolol studies including more than 500 patients reported effect sizes of about 6 mmHg. More of the smaller studies than represented on the funnel plot should report effect sizes of less than 6 mmHg.

## Discussion

The authors wish to stress that the investigated medical interventions were chosen arbitrarily based on the criteria stated in the methods section. We chose to conduct our investigation using pharmaceutical interventions for methodological reasons, but our theory is not limited to drug therapies.

### Reported effect size decreases over time

Our empirical evaluation of 206 randomised controlled trials shows that the reported effect size of three of the four investigated pharmaceuticals decreased significantly over time. When Pravastatin, Timolol and Latanoprost were new, studies reported them to be more effective than studies that were conducted in later years. We refer to this as "fading of reported effectiveness".

To many clinicians this phenomenon may induce the impression that the "true" clinical improvement is not that profound as suggested in medical publications. For example in 1978, when the anti-glaucoma beta-blocker Timolol was new, it was reported to lower the intraocular pressure (IOP) by an average of 8.17 mmHg (calculated from Figure 2). By1995, this figure decreased to an average of 6.25 mmHg. More recently, the prostaglandin-analogon Latanoprost was introduced in glaucoma therapy. In 1995, Latanoprost was reported to lower the IOP by an average of 8.25 mmHg. Compared to Timolol in the same year, Latanoprost was 2.00 mmHg more effective; compared to Timolol in 1978, Latanoprost was equally effective. Improvement may have been more a matter of perception than reality.

For one of the investigated pharmaceuticals there was no significant change of the reported effectiveness over time. For Atorvastatin analyses in relation to time may not yet be feasible as the pharmaceutical is relatively new, and there is very little variability of the publication date (1999.40 ± 1.82; Table 1).

When conducting different trials about the same topic, perfect consistency of the results certainly cannot be expected. Even the best designed studies may differ in several parameters, leading to a broad continuum of reported effect sizes as shown in Figure 2. Schmid et. al. [30] demonstrated previously that the observed treatment effect generally depends from the baseline (or control) value. This finding is expected, but a temporal trend in the development of the continuum as described above must be the result of other factors.

We are aware of methodological problems due to spurious regression when baseline values are correlated with effect size. This however does not affect the qualitative statements with respect to the correlation of baseline and effect size [29].

### Reasons for the decline of reported effectiveness

We investigated whether the decrease of the reported effectiveness was influenced by the patients' baseline level of disease, by the treatment assignment to experimental or control group or by the study size.

#### Spectrum bias (a particular form of selection bias)

For all of the investigated medical therapies we studied, the baseline values of the parameter of interest decreased over time, i. e., patients who had been included in the earlier trials were sicker than patients in later trials. This relation was highly significant for Pravastatin and Timolol and just short of the chosen level of significance for Latanoprost (Table 2 and 3). The baseline values were, again, the most important predictors of the reported effect size. Our multiple regression analysis showed that up to 80.37 percent of the effect size variability was explained by the baseline value differences (Table 4). We conclude that most of the decline of reported effectiveness over time was explained by the baseline value differences.

#### Decreasing publication bias

We found only weak evidence for the hypothesis that the decline of reported effectiveness could be mediated by study size. In theory, the combination of Publication Bias and increased study size could contribute to the gradual decrease in reported effect size. We found an increase in study size over time for Timolol (P < .0001; Table 3) and Pravastatin (P = .0517, not significant). The relation between study size and reported effect size was weak. It was significant only for Pravastatin and, then, only when the outcome variable was measured in absolute terms (P = .0139). We conclude that very little of the decrease of reported effectiveness was influenced by the study size.

#### "Shift of treatment group" bias

We did not find evidence for the hypothesis that the treatment assignment to experimental or control group influenced its reported effect size, even though there was a strong correlation between publication year and treatment group (Table 3). The latter correlation was to be expected since a medical therapy would be typically studied as the experimental therapy when new, and as the control therapy when established. The relation between treatment group and effect size was very weak. The correlation was significant only for Atorvastatin and only if the treatment effect was measured in relative terms, surprisingly favouring the *control group*. Nevertheless, the treatment group is involved in several of the best multiple regression analysis models (Table 4). These results must be interpreted with care because of the problem of multi-colinearity, especially between publication year and treatment group.

### Potential other influencing factors

Our study was limited in that we did not explore if parameters other than baseline value, treatment group and study size contribute to the decrease of the reported effect size over time. From the statistical view, there must be other factors that play a role in the temporal development of the reported effect size.

The influence of time of publication bias, study quality, and financial conflicts of interest on study outcome are known, but, to the best of our knowledge, it has not yet been studied how temporal trends of these factors influence the reported effectiveness of medical therapies over time.

The "time of publication bias", that has been described in recent years, leads to an apparently decreasing effect size. Several reports indicate that studies with positive or significant results are published on average two to three years more rapidly than studies with negative or non-significant results [10-12]. During the first years of a new pharmaceutical being available, while the publication of studies with negative results is delayed, studies with positive outcomes will dominate in meta-analyses; the size of the treatment effect may thus be overestimated. Little by little, the average reported effect size will decrease to a lower level when studies with negative results are also published. In future meta-analyses, this bias could be addressed by taking into account the date of study completion and not the date of publication.

Changes in study quality may be related to the decrease in reported effect size. During the last decades methodological trial quality has improved significantly in many areas of medicine [13,14]. There are a substantial number of reports that higher study quality is associated with lower estimates of treatment effects [15-19]. This may contribute to our observation that the reported effectiveness of medical therapies fades over time. In our meta-analysis we did not assess study quality because of the well-known lack of established quality scores [20,21], and because it is often impossible to distinguish study quality from reporting quality. Nevertheless, future meta-analyses should take into consideration the effect of trial quality development.

Unlike the other described factors, the problem of financial conflicts of interest on the part of scientists is likely to lead to an *increase *in reported effectiveness over time. In our meta-analysis this effect may have mitigated the size of the observed decrease in reported effect size. In the United States, industry's share of total investment in biomedical research and development grew from approximately 32 percent in 1980 to 62 percent in 2000 [22], and more and more industry sponsorship is being reported in many areas of medicine [13,23,24]. It is well known in the literature that studies funded by for-profit-organizations are more likely to recommend the experimental therapy as treatment of choice and less likely to report unfavourable conclusions [22,25-27]. So the reported effectiveness could improve with time. We did not investigate the role of competing financial interests in our meta-analysis because we could not determine which authors adhered to the disclosure guidelines [24,28], but further studies should address this issue.

### Consequences for the validity of meta-analyses

This study suggests that the effectiveness of medical therapies, as reported in RCTs, is not necessarily constant but that it may decline with time. A meta-analysis sums up evidence from a high number of RCTs that are conducted usually over an extensive period of time. If fading of reported effectiveness is present, the result of a meta-analysis depends on when it was performed:

(1) A meta-analysis investigating the effectiveness of one single medical therapy that was conducted when the therapy was relatively new may estimate higher treatment effects than a meta-analysis that was conducted later.

(2) A meta-analysis comparing the effectiveness of two or more different medical therapies may come to distorted results favouring the newer therapies.

We conclude that the validity of a meta-analysis where the described phenomenon is present may be impaired. In order to establish a maximum of transparency, we propose to include a test for this phenomenon in future meta-analyses. In our view it would be sufficient to plot effect size against publication year, as shown in Figure 2, and to calculate the significance level and the equation of the regression line of this correlation. Given this information, the reader could make up his own mind if the validity of the meta-analysis may be undermined.

## Conclusion

In conclusion, the current meta-analysis suggests that the effectiveness of medical therapies, as reported in randomised controlled trials, may decrease over time. We call this phenomenon "fading of reported effectiveness". Baseline differences could be identified as the main factor contributing to this effect; changes in study size or treatment group did not play a significant role. As the validity of a meta-analysis where the fading of reported effectiveness is present may be undermined, we propose to consider this problem in future meta-analyses.

## Competing interests

The author(s) declare that they have no competing interests.

## Authors' contributions

BTG participated in the design of the study, collected the raw data and drafted the manuscript. CW provided statistical expertise and performed the statistical analysis. FP generated the study question, supervised the study, and participated in the design of the study. All authors participated in analysis and interpretation of data and read and approved the final manuscript.

## Funding

None.

## Pre-publication history

The pre-publication history for this paper can be accessed here:



## Supplementary Material

Additional File 1 Appendix I: Index of studies included in the meta-analysis Click here for file

Additional File 2 Appendix II: Raw data of the studies included in the meta-analysis Click here for file
