# Supplementary material for: The fading of reported effectiveness. A meta-analysis of randomised controlled trials
Source: BMC Med Res Methodol. 2006 May 11;6:25. doi: 10.1186/1471-2288-6-25 (PMC1479361; doi:10.1186/1471-2288-6-25)
Supplement: Additional File 1 — Appendix I: Index of studies included in the meta-analysis [file 1471-2288-6-25-S1.doc]

**TABLES**

**Table 1.** Trial characteristics.

|  | **Pravastatin (n=64)** | | | | **Atorvastatin (n=35)** | | | |
| --- | --- | --- | --- | --- | --- | --- | --- | --- |
|  | Mean | ± SD | Min. | Max. | Mean | ± SD | Min. | Max. |
| Year of publication | 1995.28 | ± 3.46 | 1990 | 2001 | 1999.40 | ± 1.82 | 1996 | 2001 |
| Reported effect size[[1]](#footnote-2) | -29.50 | ± 4.16 | -19.0 | -39.0 | -36.07 | ± 3.70 | -28.4 | -44.2 |
| Baseline[[2]](#footnote-3) | 205.62 | ± 47.78 | 134.4 | 344.0 | 198.05 | ± 35.05 | 143.0 | 340.3 |
| Study size[[3]](#footnote-4) | 595.77 | ± 1511.59 | 10 | 9014 | 328.71 | ± 686.64 | 22 | 3916 |
| Treatment group[[4]](#footnote-5) | EG: 56 (87%); CG: 8 (13%) | | | | EG: 31 (89%); CG: 4 (11%) | | | |

|  | **Timolol (n=75)** | | | | **Latanoprost (n=32)** | | | |
| --- | --- | --- | --- | --- | --- | --- | --- | --- |
|  | Mean | ± SD | Min. | Max. | Mean | ± SD | Min. | Max. |
| Year of publication | 1992.68 | ± 6.99 | 1978 | 2001 | 1999.06 | ± 2.00 | 1995 | 2001 |
| Reported effect size | -6.55 | ± 1.56 | -3.65 | -11.3 | -6.83 | ± 1.53 | -3.5 | -9.8 |
| Baseline | 25.94 | ± 2.49 | 20.8 | 38.7 | 24.07 | ± 2.13 | 19.3 | 28.2 |
| Study size | 197.96 | ± 249.59 | 12 | 1198 | 152.81 | ± 195.57 | 20 | 829 |
| Treatment group | EG: 12 (16%); CG: 63 (84%) | | | | EG: 29 (91%); CG: 3 (9%) | | | |

**Table 2.** Analyses of all investigated parameters in dependence of year of publication. All given data are calculated from the equations of the regression lines. For P‑values see table 3.

|  | | **Pravastatin** | **Atorvastatin** | **Timolol** | **Latanoprost** |
| --- | --- | --- | --- | --- | --- |
| Reported effect size[[5]](#footnote-6) | Change in 5 years | -3.22 | +0.31 | -0.56 | -1.78 |
|  | 95% CI limits | (-4.50 / -1.93) | (-3.29 / +3.91) | (-0.79 / -0.34) | (-3.04 / -0.51) |
| Baseline[[6]](#footnote-7) | Change in 5 years | -41.80 | -14.63 | -0.70 | -1.82 |
|  | 95% CI limits | (-55.74 / -27.86) | (-48.38 / +19.11) | (-1.08 / -0.32) | (-3.69 / +0.05) |
| Study size[[7]](#footnote-8) | Change in 5 years | +533.54 | +233.63 | +80.55 | -16.94 |
|  | 95% CI limits | (-3.94 / +1071.01) | (-429.97 / +897.23) | (+43.38 / +117.71) | (-199.23 / +165.35) |
| Treatment group[[8]](#footnote-9) | Change in 5 years | -0.20 | -0.28 | -0.12 | -0.23 |
|  | 95% CI limits | (-0.31 / -0.08) | (-0.58 / +0.01) | (-0.17 / -0.06) | (-0.50 / +0.03) |

**Table 3.** Bivariate qualitative analyses of all investigated parameters. Significant correlations (P <0.05) are highlighted with grey background.

|  | Reported effect size[[9]](#footnote-10) | | | | Baseline | | | Study size | | | Treatment group[[10]](#footnote-11) | | |
| --- | --- | --- | --- | --- | --- | --- | --- | --- | --- | --- | --- | --- | --- |
|  | Ph | R[[11]](#footnote-12) | | P-value | Ph | R | P-value | Ph | R | P-value | Ph | R | P-value |
| Year of Publication | P: | -0.5360 | <.0001 | | P: | -0.6056 | <.0001 | P: | 0.2444 | 0.0517 | P: | -0.4092 | 0.0008 |
|  | A: | 0.0305 | 0.8618 | | A: | -0.1518 | 0.3840 | A: | 0.1237 | 0.4789 | A: | -0.3207 | 0.0603 |
|  | T: | -0.5043 | <.0001 | | T: | -0.3955 | 0.0004 | T: | 0.4512 | <.0001 | T: | -0.4513 | <.0001 |
|  | L: | -0.4642 | 0.0074 | | L: | -0.3413 | 0.0559 | L: | -0.0346 | 0.8508 | L: | -0.3167 | 0.0774 |
| Reported effect size | 1.00 | | | | P: | 0.2591 | 0.0387 | P: | -0.1899 | 0.1327 | P: | 0.1598 | 0.2073 |
|  |  |  |  |  | A: | -0.1454 | 0.4045 | A: | 0.0381 | 0.8281 | A: | -0.4339 | 0.0092 |
|  |  |  |  |  | T: | 0.6949 | <.0001 | T: | -0.1364 | 0.2432 | T: | 0.1565 | 0.1801 |
|  |  |  |  |  | L: | 0.8745 | <.0001 | L: | 0.2206 | 0.2251 | L: | 0.0289 | 0.8753 |
| Baseline |  | | | | 1.00 | | | P: | -0.2897 | 0.0202 | P: | 0.0872 | 0.4933 |
|  |  |  |  |  |  |  |  | A: | -0.0868 | 0.6201 | A: | 0.0848 | 0.6282 |
|  |  |  |  |  |  |  |  | T: | -0.1923 | 0.0983 | T: | 0.0686 | 0.5587 |
|  |  |  |  |  |  |  |  | L: | 0.2673 | 0.1392 | L: | -0.1069 | 0.5604 |
| Study size |  | | | |  | | | 1.00 | | | P: | 0.0566 | 0.6568 |
|  |  |  |  |  |  |  |  |  |  |  | A: | 0.1106 | 0.5269 |
|  |  |  |  |  |  |  |  |  |  |  | T: | -0.2723 | 0.0181 |
|  |  |  |  |  |  |  |  |  |  |  | L: | -0.3557 | 0.0457 |

**Table 4.** Multiple regression analysis to explain the variability of the parameter “reported effect size”. Shown are the top four models taking into account one or two variables with the effect size measured in absolute or in relative dimensions.

|  | Ranking of model | Effect size measured in absolute terms | | Effect size measured in relative terms | |
| --- | --- | --- | --- | --- | --- |
|  |  | Variables in model | R²[[12]](#footnote-13) | Variables in model | R² |
| **Pravastatin** | 1 | B, Y | 0.8477 | Y, B | 0.2941 |
|  | 2 | B, T | 0.8077 | Y, T | 0.2916 |
|  | 3 | B, n | 0.8060 | Y, n | 0.2910 |
|  | 4 | B | 0.8037 | Y | 0.2873 |
| **Atorvastatin** | 1 | B, T | 0.7473 | T, Y | 0.2014 |
|  | 2 | B, Y | 0.6961 | T, B | 0.2001 |
|  | 3 | B, n | 0.6960 | T, n | 0.1957 |
|  | 4 | B | 0.6959 | T | 0.1882 |
| **Timolol** | 1 | B, Y | 0.5453 | Y, B | 0.2351 |
|  | 2 | B, T | 0.4948 | Y, n | 0.2206 |
|  | 3 | B, n | 0.4829 | Y, T | 0.1979 |
|  | 4 | B | 0.4829 | Y | 0.1958 |
| **Latanoprost** | 1 | B, Y | 0.7958 | B, Y | 0.5636 |
|  | 2 | B, T | 0.7798 | B, T | 0.5306 |
|  | 3 | B, n | 0.7649 | B, n | 0.5030 |
|  | 4 | B | 0.7647 | B | 0.5030 |

1. Abbreviations: n, number of trials that met selection criteria; SD, standard deviation; EG, experimental group; CG, control group.

   ? Unit of measurement for reported effect size: Change of intraocular pressure measured in mmHg (Timolol, Latanoprost), change in low-density lipoprotein cholesterol measured in % (Pravastatin, Atorvastatin). [↑](#footnote-ref-2)
2. Unit of measurement for baseline: Intraocular pressure measured in mmHg (Timolol, Latanoprost), low-density lipoprotein cholesterol measured in mg/dl (Pravastatin, Atorvastatin). To convert low-density lipoprotein cholesterol from milligrams per deciliter to millimoles per liter, multiply milligrams per deciliter by 0.0259. [↑](#footnote-ref-3)
3. Unit of measurement for study size: Number of patients included in final analysis. [↑](#footnote-ref-4)
4. As the variable “treatment group” is either “experimental group” or “control group”, exact frequencies and percentages are given. [↑](#footnote-ref-5)
5. Abbreviations: CI, confidence interval; LDL-C, low-density lipoprotein cholesterol.

   ? Unit of measurement for reported effect size: Change of intraocular pressure measured in mmHg (Timolol, Latanoprost), change in low-density lipoprotein cholesterol measured in % (Pravastatin, Atorvastatin). [↑](#footnote-ref-6)
6. Unit of measurement for baseline: Intraocular pressure measured in mmHg (Timolol, Latanoprost), low-density lipoprotein cholesterol measured in mg/dl (Pravastatin, Atorvastatin). To convert low-density lipoprotein cholesterol from milligrams per deciliter to millimoles per liter, multiply milligrams per deciliter by 0.0259. [↑](#footnote-ref-7)
7. Unit of measurement for study size: Number of patients included in final analysis. [↑](#footnote-ref-8)
8. The parameter treatment group has two possibilities: control group=0, experimental group=1. Point biserial correlation was used to obtain the equation of the regression line and to calculate the given data. [↑](#footnote-ref-9)
9. Abbreviations: Ph, pharmaceutical; T, Timolol; L, Latanoprost; P, Pravastatin; A, Atorvastatin.

   ? Reported effect size for Pravastatin and Atorvastatin is measured in relative terms (%), for Timolol and Latanoprost in absolute terms (mmHg). [↑](#footnote-ref-10)
10. As the parameter treatment group only has the possibilities control group (=0) or experimental group (=1), point biserial correlation was used. [↑](#footnote-ref-11)
11. R: Pearson correlation coefficient. [↑](#footnote-ref-12)
12. Abbreviations: Y, year of publication; B, baseline of parameter of interest; T, treatment group; n, study size.

    ? R²: Determination coefficient. [↑](#footnote-ref-13)
