# Supplementary material for: The fading of reported effectiveness. A meta-analysis of randomised controlled trials
Source: BMC Med Res Methodol. 2006 May 11;6:25. doi: 10.1186/1471-2288-6-25 (PMC1479361; doi:10.1186/1471-2288-6-25)
Supplement: Additional File 2 — Appendix II: Raw data of the studies included in the meta-analysis [file 1471-2288-6-25-S2.doc]

#### APPENDIX

### [to be published e. g. as electronic appendix]

### I) Index of studies included in the meta-analysis

## II) Raw data of the studies included in the meta-analysis

### I) Index of studies included in the meta-analysis

### I a) Pravastatin

1. Lemay A, Dodin S, Turcot L, Dechene F, Forest JC: Estrogen/progesterone replacement versus pravastatin and their sequential association in hypercholesterolemic postmenopausal women. *Maturitas* 2001;40:247-257.

2. Stein DT, Devaraj S, Balis D, Adams-Huet B, Jialal I: Effect of statin therapy on remnant lipoprotein cholesterol levels in patients with combined hyperlipidemia. *Arterioscler Thromb Vasc Biol* 2001;21:2026-2031.

3. Isaacsohn J, Insull WJ, Stein E, et al: Long-term efficacy and safety of cerivastatin 0.8 mg in patients with primary hypercholesterolemia. *Clin Cardiol* 2001;24:IV1-IV9

4. Moyle GJ, Lloyd M, Reynolds B, Baldwin C, Mandalia S, Gazzard BG: Dietary advice with or without pravastatin for the management of hypercholesterolaemia associated with protease inhibitor therapy. *AIDS* 2001;15:1503-1508.

5. Albert MA, Danielson E, Rifai N, Ridker PM: Effect of statin therapy on C-reactive protein levels: the pravastatin inflammation/CRP evaluation (PRINCE): a randomized trial and cohort study. *JAMA* 2001;286:64-70.

6. Jialal I, Stein D, Balis D, Grundy SM, Adams-Huet B, Devaraj S: Effect of hydroxymethyl glutaryl coenzyme a reductase inhibitor therapy on high sensitive C-reactive protein levels. *Circulation* 2001;103:1933-1935.

7. Tonkin AM, Colquhoun D, Emberson J, et al: Effects of pravastatin in 3260 patients with unstable angina: results from the LIPID study. *Lancet*  2000;356:1871-1875.

8. Dujovne CA, Knopp R, Kwiterovich P, Hunninghake D, McBride TA, Poland M: Randomized comparison of the efficacy and safety of cerivastatin and pravastatin in 1,030 hypercholesterolemic patients. The Cerivastatin Study Group. *Mayo Clin Proc* 2000;75:1124-1132.

9. Santos RD, Sposito AC, Ventura LI, Cesar LA, Ramires JA, Maranhao RC: Effect of pravastatin on plasma removal of a chylomicron-like emulsion in men with coronary artery disease. *Am J Cardiol* 2000;85:1163-1166.

10. Rustemeijer C, Schouten JA, Voerman HJ, Hensgens HE, Donker AJ, Heine RJ: Pravastatin compared to bezafibrate in the treatment of dyslipidemia in insulin-treated patients with type 2 diabetes mellitus. *Diabetes Metab Res Rev* 2000;16:82-87.

11. Dobs AS, Miller S, Neri G, et al: Effects of simvastatin and pravastatin on gonadal function in male hypercholesterolemic patients. *Metabolism* 2000;49:115-121.

12. Seed M, Weir MR: Double-masked comparison of the quality of life of hypercholesterolemic men treated with simvastatin or pravastatin. International Quality of Life Multicenter Group. *Clin Ther* 1999;21:1758-1770.

13. Dupuis J, Tardif JC, Cernacek P, Theroux P: Cholesterol reduction rapidly improves endothelial function after acute coronary syndromes. The RECIFE (reduction of cholesterol in ischemia and function of the endothelium) trial. *Circulation* 1999;99:3227-3233.

14. Scheen AJ: [Clinical study of the month. The LIPID study: "long-term intervention with pravastatin in ischaemic disease"].  *Rev Med Liege* 1999;54:2-3.

15. Goldberg RB, Mellies MJ, Sacks FM, et al: Cardiovascular events and their reduction with pravastatin in diabetic and glucose-intolerant myocardial infarction survivors with average cholesterol levels: subgroup analyses in the cholesterol and recurrent events (CARE) trial. The Care Investigators. *Circulation* 1998;98:2513-2519.

16. Lewis SJ, Moye LA, Sacks FM, et al: Effect of pravastatin on cardiovascular events in older patients with myocardial infarction and cholesterol levels in the average range. Results of the Cholesterol and Recurrent Events (CARE) trial. *Ann Intern Med* 1998;129:681-689.

17. Contacos C, Barter PJ, Vrga L, Sullivan DR: Cholesteryl ester transfer in hypercholesterolaemia: fasting and postprandial studies with and without pravastatin. *Atherosclerosis* 1998;141:87-98.

18. Lewis SJ, Sacks FM, Mitchell JS, et al: Effect of pravastatin on cardiovascular events in women after myocardial infarction: the cholesterol and recurrent events (CARE) trial. *J Am Coll Cardiol* 1998;32:140-146.

19. MacMahon S, Sharpe N, Gamble G, et al: Effects of lowering average of below-average cholesterol levels on the progression of carotid atherosclerosis: results of the LIPID Atherosclerosis Substudy. LIPID Trial Research Group. *Circulation* 1998;97:1784-1790.

20. Eriksson M, Hadell K, Holme I, Walldius G, Kjellstrom T: Compliance with and efficacy of treatment with pravastatin and cholestyramine: a randomized study on lipid-lowering in primary care. *J Intern Med* 1998;243:373-380.

21. Jones P, Kafonek S, Laurora I, Hunninghake D: Comparative dose efficacy study of atorvastatin versus simvastatin, pravastatin, lovastatin, and fluvastatin in patients with hypercholesterolemia (the CURVES study). *Am J Cardiol* 1998;81:582-587.

22. Mostaza JM, Schulz I, Vega GL, Grundy SM: Comparison of pravastatin with crystalline nicotinic acid monotherapy in treatment of combined hyperlipidemia. *Am J Cardiol* 1997;79:1298-1301.

23. Mercuri M, Bond MG, Sirtori CR, et al: Pravastatin reduces carotid intima-media thickness progression in an asymptomatic hypercholesterolemic mediterranean population: the Carotid Atherosclerosis Italian Ultrasound Study. *Am J Med* 1996;101:627-634.

24. Sacks FM, Pfeffer MA, Moye LA, et al: The effect of pravastatin on coronary events after myocardial infarction in patients with average cholesterol levels. Cholesterol and Recurrent Events Trial investigators. *N Engl J Med* 1996;335:1001-1009.

25. Galvan AQ, Natali A, Baldi S, et al: Effect of a reduced-fat diet with or without pravastatin on glucose tolerance and insulin sensitivity in patients with primary hypercholesterolemia. *J Cardiovasc Pharmacol* 1996;28:595-602.

26. Wiklund O, Bondjers G, Wright I, Camejo G: Insoluble complex formation between LDL and arterial proteoglycans in relation to serum lipid levels and effects of lipid lowering drugs. *Atherosclerosis* 1996;119:57-67.

27. Shepherd J, Cobbe SM, Ford I, et al: Prevention of coronary heart disease with pravastatin in men with hypercholesterolemia. West of Scotland Coronary Prevention Study Group. *N Engl J Med* 1995;333:1301-1307.

28. Pitt B, Mancini GB, Ellis SG, Rosman HS, Park JS, McGovern ME: Pravastatin limitation of atherosclerosis in the coronary arteries (PLAC I): reduction in atherosclerosis progression and clinical events. PLAC I investigation. *J Am Coll Cardiol* 1995;26:1133-1139.

29. Dobs AS, Prasad M, Goldberg A, Guccione M, Hoover DR: Changes in serum lipoprotein(a) in hyperlipidemic subjects undergoing long-term treatment with lipid-lowering drugs. *Cardiovasc Drugs Ther* 1995;9:677-684.

30. Salonen R, Nyyssonen K, Porkkala E, et al: Kuopio Atherosclerosis Prevention Study (KAPS). A population-based primary preventive trial of the effect of LDL lowering on atherosclerotic progression in carotid and femoral arteries. *Circulation* 1995;92:1758-1764.

31. Jacotot B, Benghozi R, Pfister P, Holmes D: Comparison of fluvastatin versus pravastatin treatment of primary hypercholesterolemia. French Fluvastatin Study Group. *Am J Cardiol* 1995;76:54A-56A.

32. Milani M, Cimminiello C, Merlo B, Lorena M, Arpaia G, Bonfardeci G: Effects of fluvastatin and pravastatin on lipid profiles and thromboxane production in type IIa hypercholesterolemia. *Am J Cardiol* 1995;76:51A-53A.

33. Le Quan S, Levenson J, Megnien JL, Simon A, Devynck MA: Platelet cytosolic Ca2+ and membrane dynamics in patients with primary hypercholesterolemia. Effects of pravastatin. *Arterioscler Thromb Vasc Biol* 1995;15:759-764.

34. Vanhanen HT, Miettinen TA: Cholesterol absorption and synthesis during pravastatin, gemfibrozil and their combination. *Atherosclerosis* 1995;115:135-146.

35. Ordovas JM, Lopez-Miranda J, Perez-Jimenez F, et al: Effect of apolipoprotein E and A-IV phenotypes on the low density lipoprotein response to HMG CoA reductase inhibitor therapy. *Atherosclerosis* 1995;113:157-166.

36. Kool M, Lustermans F, Kragten H, et al: Does lowering of cholesterol levels influence functional properties of large arteries? *Eur J Clin Pharmacol* 1995;48:217-223.

37. Hansen PS, Meinertz H, Gerdes LU, Klausen IC, Faergeman O: Treatment of patients with familial defective apolipoprotein B-100 with pravastatin and gemfibrozil: a two-period cross-over study. *Clin Investig* 1994;72:1065-1070.

38. Giannini SD, Bertolami MC, Scholz J, Faludi AA, Forti N, Diament J: [Randomized, double-blind comparative study between pravastatin and lovastatin. Evaluation of efficacy and safety]. *Arq Bras Cardiol* 1994;63:327-332.

39. Tsalamandris C, Panagiotopoulos S, Sinha A, Cooper ME, Jerums G: Complementary effects of pravastatin and nicotinic acid in the treatment of combined hyperlipidaemia in diabetic and non-diabetic patients. *J Cardiovasc Risk* 1994;1:231-239.

40. Kostis JB, Rosen RC, Wilson AC: Central nervous system effects of HMG CoA reductase inhibitors: lovastatin and pravastatin on sleep and cognitive performance in patients with hypercholesterolemia. *J Clin Pharmacol* 1994;34:989-996.

41. Celis H, Lijnen P, Fagard R, Staessen J, Thijs L, Amery A: Efficacy and safety of pravastatin in hypertensive hypercholesterolaemic patients on antihypertensive drug therapy. *J Hum Hypertens* 1994;8:525-530.

42. Davignon J, Roederer G, Montigny M, et al: Comparative efficacy and safety of pravastatin, nicotinic acid and the two combined in patients with hypercholesterolemia. *Am J Cardiol* 1994;73:339-345.

43. Contacos C, Barter PJ, Sullivan DR: Effect of pravastatin and omega-3 fatty acids on plasma lipids and lipoproteins in patients with combined hyperlipidemia. *Arterioscler Thromb* 1993;13:1755-1762.

44. Stalenhoef AF, Lansberg PJ, Kroon AA, et al: Treatment of primary hypercholesterolaemia. Short-term efficacy and safety of increasing doses of simvastatin and pravastatin: a double-blind comparative study. *J Intern Med* 1993;234:77-82.

45. Comparative efficacy and safety of pravastatin and cholestyramine alone and combined in patients with hypercholesterolemia. Pravastatin Multicenter Study Group II. *Arch Intern Med* 1993;153:1321-1329.

46. A multicenter comparative trial of lovastatin and pravastatin in the treatment of hypercholesterolemia. The Lovastatin Pravastatin Study Group. *Am J Cardiol* 1993;71:810-815.

47. Klausen IC, Gerdes LU, Meinertz H, Hansen FA, Faergeman O: Apolipoprotein(a) polymorphism predicts the increase of Lp(a) by pravastatin in patients with familial hypercholesterolaemia treated with bile acid sequestration. *Eur J Clin Invest* 1993;23:240-245.

48. Eckernas SA, Roos BE, Kvidal P, et al: The effects of simvastatin and pravastatin on objective and subjective measures of nocturnal sleep: a comparison of two structurally different HMG CoA reductase inhibitors in patients with primary moderate hypercholesterolaemia. *Br J Clin Pharmacol* 1993;35:284-289.

49. Wiklund O, Angelin B, Bergman M, et al: Pravastatin and gemfibrozil alone and in combination for the treatment of hypercholesterolemia. *Am J Med* 1993;94:13-20.

50. Simons LA, Nestel PJ, Clifton P, Janus ED, Simons J, Parfitt A: Treatment of primary hypercholesterolaemia with pravastatin: efficacy and safety over three years. *Med J Aust* 1992;157:584-589.

51. Craveri A, Colombo L, Citella C, et al: [Changes in lipid status during pravastatin treatment]. *Minerva Med* 1992;83:695-703.

52. Betteridge DJ, Bhatnager D, Bing RF, et al: Treatment of familial hypercholesterolaemia. United Kingdom lipid clinics study of pravastatin and cholestyramine. *BMJ* 1992;304:1335-1338.

53. Gomez-Perez FJ, Bustamante F, Vergara A, Villasenor J, Wong B, Rull JA: A controlled trial of pravastatin vs probucol in the treatment of primary hypercholesterolemia. *Rev Invest Clin* 1992;44:53-61.

54. Rubenfire M, Maciejko JJ, Blevins RD, Orringer C, Kobylak L, Rosman H: The effect of pravastatin on plasma lipoprotein and apolipoprotein levels in primary hypercholesterolemia. The Southeastern Michigan Collaborative Group. *Arch Intern Med* 1991;151:2234-2240.

55. Sienra-Perez JC, Lerman-Garber I, Ahumada-Ayala M, et al: [Pravastatin vs. probucol in the treatment of hypercholesterolemia. A double-blind study]. *Arch Inst Cardiol Mex* 1991;61:365-373.

56. Reihner E, Rudling M, Stahlberg D, et al: [Effect of pravastatin on hepatic cholesterol metabolism]. *Fortschr Med* 1991;109:189-194.

57. Jones PH, Farmer JA, Cressman MD, et al: Once-daily pravastatin in patients with primary hypercholesterolemia: a dose-response study. *Clin Cardiol* 1991;14:146-151.

58. Crepaldi G, Baggio G, Arca M, et al: Pravastatin vs gemfibrozil in the treatment of primary hypercholesterolemia. The Italian Multicenter Pravastatin Study I. *Arch Intern Med* 1991;151:146-152.

59. Schwartzkopff W, Bimmermann A, Schleicher J: [Comparison of the effectiveness of the HMG-CoA-reductase inhibitors pravastatin versus colestyramine in hypercholesteremia]. *Arzneimittelforschung* 1990;40:1322-1327.

60. Hunninghake DB, Mellies MJ, Goldberg AC, et al: Efficacy and safety of pravastatin in patients with primary hypercholesterolemia. II. Once-daily versus twice-daily dosing. *Atherosclerosis* 1990;85:219-227.

61. Ismail F, Corder CN, Epstein S, Barbi G, Thomas S: Effects of pravastatin and cholestyramine on circulating levels of parathyroid hormone and vitamin D metabolites. *Clin Ther* 1990;12:427-430.

62. Hoogerbrugge N, Mol MJ, Van Dormaal JJ, et al: The efficacy and safety of pravastatin, compared to and in combination with bile acid binding resins, in familial hypercholesterolaemia. *J Intern Med* 1990;228:261-266.

63. Pan HY, DeVault AR, Swites BJ, et al: Pharmacokinetics and pharmacodynamics of pravastatin alone and with cholestyramine in hypercholesterolemia. *Clin Pharmacol Ther* 1990;48:201-*207.*

64. Vega GL, Krauss RM, Grundy SM: Pravastatin therapy in primary moderate hypercholesterolaemia: changes in metabolism of apolipoprotein B-containing lipoproteins. *J Intern Med* 1990;227:81-94.

### I b) Atorvastatin

65. Wang KY, Ting CT: A randomized, double-blind, placebo-controlled, 8-week study to evaluate the efficacy and safety of once daily atorvastatin (10 mg) in patients with elevated LDL-cholesterol. *Jpn Heart J* 2001;42:725-738.

66. Stein DT, Devaraj S, Balis D, Adams-Huet B, Jialal I: Effect of statin therapy on remnant lipoprotein cholesterol levels in patients with combined hyperlipidemia. *Arterioscler Thromb Vasc Biol* 2001;21:2026-2031.

67. Malik J, Melenovsky V, Wichterle D, et al: Both fenofibrate and atorvastatin improve vascular reactivity in combined hyperlipidaemia (fenofibrate versus atorvastatin trial--FAT). *Cardiovasc Res* 2001;52:290-298.

68. Hunninghake D, Insull WJ, Toth P, Davidson D, Donovan JM, Burke SK: Coadministration of colesevelam hydrochloride with atorvastatin lowers LDL cholesterol additively. *Atherosclerosis* 2001;158:407-416.

69. Hunninghake D, Insull W, Knopp R, et al: Comparison of the efficacy of atorvastatin versus cerivastatin in primary hypercholesterolemia. *Am J Cardiol* 2001;88:635-639.

70. Andrews TC, Ballantyne CM, Hsia JA, Kramer JH: Achieving and maintaining National Cholesterol Education Program low-density lipoprotein cholesterol goals with five statins. *Am J Med* 2001;111:185-191.

71. Olsson AG, Pears J, McKellar J, Mizan J, Raza A: Effect of rosuvastatin on low-density lipoprotein cholesterol in patients with hypercholesterolemia. *Am J Cardiol* 2001;88:504-508.

72. Pedro-Botet J, Schaefer EJ, Bakker-Arkema RG, et al: Apolipoprotein E genotype affects plasma lipid response to atorvastatin in a gender specific manner. *Atherosclerosis* 2001;158:183-193.

73. The effect of aggressive versus standard lipid lowering by atorvastatin on diabetic dyslipidemia: the DALI study: a double-blind, randomized, placebo-controlled trial in patients with type 2 diabetes and diabetic dyslipidemia. *Diabetes Care* 2001;24:1335-1341.

74. McKenney JM, McCormick LS, Schaefer EJ, Black DM, Watkins ML: Effect of niacin and atorvastatin on lipoprotein subclasses in patients with atherogenic dyslipidemia. *Am J Cardiol* 2001;88:270-274.

75. Branchi A, Fiorenza AM, Torri A, et al: Effects of low doses of simvastatin and atorvastatin on high-density lipoprotein cholesterol levels in patients with hypercholesterolemia. *Clin Ther* 2001;23:851-857.

76. Nordoy A, Hansen JB, Brox J, Svensson B: Effects of atorvastatin and omega-3 fatty acids on LDL subfractions and postprandial hyperlipemia in patients with combined hyperlipemia. *Nutr Metab Cardiovasc Dis* 2001;11:7-16.

77. Jialal I, Stein D, Balis D, Grundy SM, Adams-Huet B, Devaraj S: Effect of hydroxymethyl glutaryl coenzyme a reductase inhibitor therapy on high sensitive C-reactive protein levels. *Circulation* 2001;103:1933-1935.

78. Insull W, Kafonek S, Goldner D, Zieve F: Comparison of efficacy and safety of atorvastatin (10mg) with simvastatin (10mg) at six weeks. ASSET Investigators. *Am J Cardiol* 2001;87:554-559.

79. Gentile S, Turco S, Guarino G, et al: Comparative efficacy study of atorvastatin vs simvastatin, pravastatin, lovastatin and placebo in type 2 diabetic patients with hypercholesterolaemia. *Diabetes Obes Metab* 2000;2:355-362.

80. Renders L, Mayer-Kadner I, Koch C, et al: Efficacy and drug interactions of the new HMG-CoA reductase inhibitors cerivastatin and atorvastatin in CsA-treated renal transplant recipients. *Nephrol Dial Transplant* 2001;16:141-146.

81. Giral P, Bruckert E, Jacob N, Chapman MJ, Foglietti MJ, Turpin G: Homocysteine and lipid lowering agents. A comparison between atorvastatin and fenofibrate in patients with mixed hyperlipidemia. *Atherosclerosis* 2001;154:421-427.

82. Recto CS, Acosta S, Dobs A: Comparison of the efficacy and tolerability of simvastatin and atorvastatin in the treatment of hypercholesterolemia. *Clin Cardiol* 2000;23:682-688.

83. Schrott HG, Knapp H, Davila M, Shurzinske L, Black D: Effect of atorvastatin on blood lipid levels in the first 2 weeks of treatment: a randomized, placebo-controlled study. *Am Heart J* 2000;140:249-252.

84. Farnier M, Portal JJ, Maigret P: Efficacy of atorvastatin compared with simvastatin in patients with hypercholesterolemia.  *J Cardiovasc Pharmacol Ther* 2000;5:27-32.

85. Branchi A, Fiorenza AM, Rovellini A, et al: Lowering effects of four different statins on serum triglyceride level. *Eur J Clin Pharmacol* 1999;55:499-502.

86. Bairaktari ET, Tzallas CS, Tsimihodimos VK, Liberopoulos EN, Miltiadous GA, Elisaf MS: Comparison of the efficacy of atorvastatin and micronized fenofibrate in the treatment of mixed hyperlipidemia. *J Cardiovasc Risk* 1999;6:113-116.

87. Hunninghake D, Bakker-Arkema RG, Wigand JP, et al: Treating to meet NCEP-recommended LDL cholesterol concentrations with atorvastatin, fluvastatin, lovastatin, or simvastatin in patients with risk factors for coronary heart disease. *J Fam Pract* 1998;47:349-356.

88. Brown AS, Bakker-Arkema RG, Yellen L, et al: Treating patients with documented atherosclerosis to National Cholesterol Education Program-recommended low-density-lipoprotein cholesterol goals with atorvastatin, fluvastatin, lovastatin and simvastatin. *J Am Coll Cardiol* 1998;32:665-672.

89. Simons LA: Comparison of atorvastatin alone versus simvastatin +/- cholestyramine in the management of severe primary hypercholesterolaemia (the six cities study). *Aust N Z J Med* 1998;28:327-333.

90. McKenney JM, McCormick LS, Weiss S, Koren M, Kafonek S, Black DM: A randomized trial of the effects of atorvastatin and niacin in patients with combined hyperlipidemia or isolated hypertriglyceridemia. Collaborative Atorvastatin Study Group. *Am J Med* 1998;104:137-143.

91. Jones P, Kafonek S, Laurora I, Hunninghake D: Comparative dose efficacy study of atorvastatin versus simvastatin, pravastatin, lovastatin, and fluvastatin in patients with hypercholesterolemia (the CURVES study). *Am J Cardiol* 1998;81:582-587.

92. Ooi TC, Heinonen T, Alaupovic P, et al: Efficacy and safety of a new hydroxymethylglutaryl-coenzyme A reductase inhibitor, atorvastatin, in patients with combined hyperlipidemia: comparison with fenofibrate. *Arterioscler Thromb Vasc Biol* 1997;17:1793-1799.

93. Davidson MH, Nawrocki JW, Weiss SR, et al: Effectiveness of atorvastatin for reducing low-density lipoprotein cholesterol to National Cholesterol Education Program treatment goals. *Am J Cardiol* 1997;80:347-348.

94. Dart A, Jerums G, Nicholson G, et al: A multicenter, double-blind, one-year study comparing safety and efficacy of atorvastatin versus simvastatin in patients with hypercholesterolemia. *Am J Cardiol* 1997;80:39-44.

95. Davidson M, McKenney J, Stein E, et al: Comparison of one-year efficacy and safety of atorvastatin versus lovastatin in primary hypercholesterolemia. Atorvastatin Study Group I. *Am J Cardiol* 1997;79:1475-1481.

96. Efficacy of atorvastatin in primary hypercholesterolemia. Japan Cholesterol Lowering Atorvastatin Study (J-CLAS) Group. *Am J Cardiol* 1997;79:1248-1252.

97. Bertolini S, Bon GB, Campbell LM, et al: Efficacy and safety of atorvastatin compared to pravastatin in patients with hypercholesterolemia. *Atherosclerosis* 1997;130:191-197.

98. Heinonen TM, Stein E, Weiss SR, et al: The lipid-lowering effects of atorvastatin, a new HMG-CoA reductase inhibitor: results of a randomized, double-masked study. *Clin Ther* 1996;18:853-863.

99. Nawrocki JW, Weiss SR, Davidson MH, et al: Reduction of LDL cholesterol by 25% to 60% in patients with primary hypercholesterolemia by atorvastatin, a new HMG-CoA reductase inhibitor. *Arterioscler Thromb Vasc Biol* 1995;15:678-682.

### I c) Timolol

100. Netland PA, Landry T, Sullivan EK, et al: Travoprost compared with latanoprost and timolol in patients with open-angle glaucoma or ocular hypertension. *Am J Ophthalmol* 2001;132:472-484.

101. Goldberg I, Cunha-Vaz J, Jakobsen JE, Nordmann JP, Trost E, Sullivan EK: Comparison of topical travoprost eye drops given once daily and timolol 0.5% given twice daily in patients with open-angle glaucoma or ocular hypertension. *J Glaucoma* 2001;10:414-422.

102. Laibovitz RA, VanDenburgh AM, Felix C, et al: Comparison of the ocular hypotensive lipid AGN 192024 with timolol: dosing, efficacy, and safety evaluation of a novel compound for glaucoma management. *Arch Ophthalmol* 2001;119:994-1000.

103. Brandt JD, VanDenburgh AM, Chen K, Whitcup SM: Comparison of once- or twice-daily bimatoprost with twice-daily timolol in patients with elevated IOP : a 3-month clinical trial. *Ophthalmology* 2001;108:1023-1031.

104. Sherwood M, Brandt J: Six-month comparison of bimatoprost once-daily and twice-daily with timolol twice-daily in patients with elevated intraocular pressure. *Surv Ophthalmol* 2001;45 Suppl 4:S361-S368

105. Shedden A, Laurence J, Tipping R: Efficacy and tolerability of timolol maleate ophthalmic gel-forming solution versus timolol ophthalmic solution in adults with open-angle glaucoma or ocular hypertension: a six-month, double-masked, multicenter study. *Clin Ther* 2001;23:440-450.

106. Lubeck P, Orgul S, Gugleta K, Gherghel D, Gekkieva M, Flammer J: Effect of timolol on anterior optic nerve blood flow in patients with primary open-angle glaucoma as assessed by the Heidelberg retina flowmeter. *J Glaucoma* 2001;10:13-17.

107. Shimazaki J, Hanada K, Yagi Y, et al: Changes in ocular surface caused by antiglaucomatous eyedrops: prospective, randomised study for the comparison of 0.5% timolol v 0. 12% unoprostone. *Br J Ophthalmol* 2000;84:1250-1254.

108. Heijl A, Bengtsson B: Long-term effects of timolol therapy in ocular hypertension: a double-masked, randomised trial. *Graefes Arch Clin Exp Ophthalmol* 2000;238:877-883.

109. Orzalesi N, Rossetti L, Invernizzi T, Bottoli A, Autelitano A: Effect of timolol, latanoprost, and dorzolamide on circadian IOP in glaucoma or ocular hypertension. *Invest Ophthalmol Vis Sci* 2000;41:2566-2573.

110. Javitt JC, Schiffman RM: Clinical success and quality of life with brimonidine 0.2% or timolol 0.5% used twice daily in glaucoma or ocular t hypertension: a randomized clinical trial. Brimonidine Outcomes Study Group I. *J Glaucoma* 2000;9:224-234.

111. Aung T, Wong HT, Yip CC, Leong JY, Chan YH, Chew PT: Comparison of the intraocular pressure-lowering effect of latanoprost and timolol in patients with chronic angle closure glaucoma: a preliminary study. *Ophthalmology* 2000;107:1178-1183.

112. Hedman K, Alm A: A pooled-data analysis of three randomized, double-masked, six-month clinical studies comparing the intraocular pressure reducing effect of latanoprost and timolol. *Eur J Ophthalmol* 2000;10:95-104.

113. March WF, Ochsner KI: The long-term safety and efficacy of brinzolamide 1.0% (azopt) in patients with primary open-angle glaucoma or ocular hypertension. The Brinzolamide Long-Term Therapy Study Group.  *Am J Ophthalmol* 2000;129:136-143.

114. Mirza GE, Karakucuk S, Temel E: Comparison of the effects of 0.5% timolol maleate, 2% carteolol hydrochloride, and 0.3% metipranolol on intraocular pressure and perimetry findings and evaluation of their ocular and systemic effects. *J Glaucoma* 2000;9:45-50.

115. Melamed S, David R: Ongoing clinical assessment of the safety profile and efficacy of brimonidine compared with timolol: year-three results. Brimonidine Study Group II. *Clin Ther* 2000;22:103-111.

116. Konstas AG, Maltezos AC, Gandi S, Hudgins AC, Stewart WC: Comparison of 24-hour intraocular pressure reduction with two dosing regimens of latanoprost and timolol maleate in patients with primary open-angle glaucoma. *Am J Ophthalmol* 1999;128:15-20.

117. Mastropasqua L, Carpineto P, Ciancaglini M, Gallenga PE: A 12-month, randomized, double-masked study comparing latanoprost with timolol in pigmentary glaucoma. *Ophthalmology* 1999;106:550-555.

118. Katz LJ: Brimonidine tartrate 0.2% twice daily vs timolol 0.5% twice daily: 1-year results in glaucoma patients. Brimonidine Study Group. *Am J Ophthalmol* 1999;127:20-26.

119. Schenker H, Maloney S, Liss C, Gormley G, Hartenbaum D: Patient preference, efficacy, and compliance with timolol maleate ophthalmic gel-forming solution versus timolol maleate ophthalmic solution in patients with ocular hypertension or open-angle glaucoma. *Clin Ther* 1999;21:138-147.

120. Stewart WC, Stewart JA, Kapik BM: The effects of unoprostone isopropyl 0.12% and timolol maleate 0.5% on diurnal intraocular pressure. *J Glaucoma* 1998;7:388-394.

121. Boyle JE, Ghosh K, Gieser DK, Adamsons IA: A randomized trial comparing the dorzolamide-timolol combination given twice daily to monotherapy with timolol and dorzolamide. Dorzolamide-Timolol Study Group. *Ophthalmology* 1998;105:1945-1951.

122. LeBlanc RP: Twelve-month results of an ongoing randomized trial comparing brimonidine tartrate 0.2% and timolol 0.5% given twice daily in patients with glaucoma or ocular hypertension. Brimonidine Study Group 2. *Ophthalmology* 1998;105:1960-1967.

123. Camras CB, Wax MB, Ritch R, et al: Latanoprost treatment for glaucoma: effects of treating for 1 year and of switching from timolol. United States Latanoprost Study Group. *Am J Ophthalmol* 1998;126:390-399.

124. Silver LH: Clinical efficacy and safety of brinzolamide (Azopt), a new topical carbonic anhydrase inhibitor for primary open-angle glaucoma and ocular hypertension. Brinzolamide Primary Therapy Study Group. *Am J Ophthalmol* 1998;126:400-408.

125. Watson PG: Latanoprost. Two years' experience of its use in the United Kingdom. Latanoprost Study Group. *Ophthalmology* 1998;105:82-87.

126. Stewart WC, Cohen JS, Netland PA, Weiss H, Nussbaum LL: Efficacy of carteolol hydrochloride 1% vs timolol maleate 0.5% in patients with increased intraocular pressure. Nocturnal Investigation of Glaucoma Hemodynamics Trial Study Group. *Am J Ophthalmol* 1997;124:498-505.

127. Schuman JS, Horwitz B, Choplin NT, David R, Albracht D, Chen K: A 1-year study of brimonidine twice daily in glaucoma and ocular hypertension. A controlled, randomized, multicenter clinical trial. Chronic Brimonidine Study Group. *Arch Ophthalmol* 1997;115:847-852.

128. Yamamoto T, Kitazawa Y, Azuma I, Masuda K: Clinical evaluation of UF-021 (Rescula; isopropyl unoprostone). *Surv Ophthalmol* 1997;41 Suppl 2:S99-103.

129. Diestelhorst M, Roters S, Krieglstein GK: The effect of latanoprost 0.005% once daily versus 0.0015% twice daily on intraocular pressure and aqueous humour protein concentration in glaucoma patients. A randomized, double-masked comparison with timolol 0.5%. *Graefes Arch Clin Exp Ophthalmol* 1997;235:20-26.

130. Schuman JS: Clinical experience with brimonidine 0.2% and timolol 0.5% in glaucoma and ocular hypertension. *Surv Ophthalmol* 1996;41 Suppl 1:S27-S37

131. Mishima HK, Masuda K, Kitazawa Y, Azuma I, Araie M: A comparison of latanoprost and timolol in primary open-angle glaucoma and ocular hypertension. A 12-week study. *Arch Ophthalmol* 1996;114:929-932.

132. Stewart WC, Laibovitz R, Horwitz B, Stewart RH, Ritch R, Kottler M: A 90-day study of the efficacy and side effects of 0.25% and 0.5% apraclonidine vs 0.5% timolol. Apraclonidine Primary Therapy Study Group. *Arch Ophthalmol* 1996;114:938-942.

133. Dubiner HB, Hill R, Kaufman H, et al: Timolol hemihydrate vs timolol maleate to treat ocular hypertension and open-angle glaucoma. *Am J Ophthalmol* 1996;121:522-528.

134. Rosenlund EF: The intraocular pressure lowering effect of timolol in gel-forming solution. *Acta Ophthalmol Scand* 1996;74:160-162.

135. Fristrom B: A 6-month, randomized, double-masked comparison of latanoprost with timolol in patients with open angle glaucoma or ocular hypertension. *Acta Ophthalmol Scand* 1996;74:140-144.

136. Simpson AJ, Gray TB, Ballantyne C: A controlled clinical trial of dorzolamide: a single-centre subset of a multicentre study. *Aust N Z J Ophthalmol* 1996;24:39-42.

137. Watson P, Stjernschantz J: A six-month, randomized, double-masked study comparing latanoprost with timolol in open-angle glaucoma and ocular hypertension. The Latanoprost Study Group. *Ophthalmology* 1996;103:126-137.

138. Camras CB: Comparison of latanoprost and timolol in patients with ocular hypertension and glaucoma: a six-month masked, multicenter trial in the United States. The United States Latanoprost Study Group. *Ophthalmology* 1996;103:138-147.

139. Alm A, Stjernschantz J: Effects on intraocular pressure and side effects of 0.005% latanoprost applied once daily, evening or morning. A comparison with timolol. Scandinavian Latanoprost Study Group. *Ophthalmology* 1995;102:1743-1752.

140. Strahlman E, Tipping R, Vogel R: A double-masked, randomized 1-year study comparing dorzolamide (Trusopt), timolol, and betaxolol. International Dorzolamide Study Group. *Arch Ophthalmol* 1995;113:1009-1016.

141. Akafo SK, Thompson JR, Rosenthal AR: A cross-over trial comparing once daily levobunolol with once and twice daily timolol. *Eur J Ophthalmol* 1995;5:172-176.

142. Nagasubramanian S, Hitchings RA, Demailly P, et al: Comparison of apraclonidine and timolol in chronic open-angle glaucoma. A three-month study. *Ophthalmology* 1993;100:1318-1323.

143. Azuma I, Masuda K, Kitazawa Y, Takase M, Yamamura H: Double-masked comparative study of UF-021 and timolol ophthalmic solutions in patients with primary open-angle glaucoma or ocular hypertension. *Jpn J Ophthalmol* 1993;37:514-525.

144. Flammer J, Kitazawa Y, Bonomi L, et al: Influence of carteolol and timolol on IOP an visual fields in glaucoma: a multi-center, double-masked, prospective study. *Eur J Ophthalmol* 1992;2:169-174.

145. Stewart WC, Shields MB, Allen RC, et al: A 3-month comparison of 1% and 2% carteolol and 0.5% timolol in open-angle glaucoma. *Graefes Arch Clin Exp Ophthalmol* 1991;229:258-261.

146. Levobunolol. A four-year study of efficacy and safety in glaucoma treatment. The Levobunolol Study Group. *Ophthalmology* 1989;96:642-645.

147. Geyer O, Lazar M, Novack GD, Shen D, Eto CY: Levobunolol compared with timolol: a four-year study. *Br J Ophthalmol* 1988;72:892-896.

148. Freyler H, Novack GD, Menapace R, Skorpik C, Mordaunt J, Batoosingh AL: [Comparison of the effectiveness and safety of levobunolol and timolol in ocular hypertension and chronic open-angle glaucoma]. *Klin Monatsbl Augenheilkd* 1988;193:257-260.

149. Seamone C, LeBlanc R, Saheb N, Novack G: Efficacy of twice-daily levobunolol in the treatment of elevated intraocular pressure. *Can J Ophthalmol* 1988;23:168-170.

150. Feghali JG, Kaufman PL, Radius RL, Mandell AI: A comparison of betaxolol and timolol in open angle glaucoma and ocular hypertension. *Acta Ophthalmol (Copenh)* 1988;66:180-186.

151. Alexander DW, Berson FG, Epstein DL: A clinical trial of timolol and epinephrine in the treatment of primary open-angle glaucoma. *Ophthalmology* 1988;95:247-251.

152. Savelsbergh-Fillette MP, Demailly P: [Comparative study of levobunolol and timolol in the treatment of chronic open-angle glaucoma and chronic ocular hypertension]. *J Fr Ophtalmol* 1988;11:587-590.

153. Elman J, Caprioli J, Rosanelli EGJ, et al: Celiprolol versus timolol and placebo: a two week double-blind comparison. *J Ocul Pharmacol* 1987;3:5-10.

154. Geyer O, Lazar M, Novack GD, Lue JC, Duzman E: Levobunolol compared with timolol for the control of elevated intraocular pressure. *Ann Ophthalmol* 1986;18:289-90, 292.

155. Tsoy EA, Meekins BB, Shields MB: Comparison of two treatment schedules for combined timolol and dipivefrin therapy. *Am J Ophthalmol* 1986;102:320-324.

156. Stewart RH, Kimbrough RL, Ward RL: Betaxolol vs timolol. A six-month double-blind comparison. *Arch Ophthalmol* 1986;104:46-48.

157. Uusitalo RJ, Palkama A, Stjernschantz J: A study of the efficacy of two commercial preparations of timolol maleate with special reference to side effects. *Acta Ophthalmol (Copenh)* 1985;63:634-641.

158. Stryz JR, Merte HJ: [Pressure lowering effect and side effects of 0.5% and 1.0% levobunolol eyedrops, compared with 0.5% timolol eyedrops in patients with open-angle glaucoma]. *Klin Monatsbl Augenheilkd* 1985;187:537-544.

159. Levobunolol. A beta-adrenoceptor antagonist effective in the long-term treatment of glaucoma. The Levobunolol Study Group (Appended). *Ophthalmology* 1985;92:1271-1276.

160. Ober M, Scharrer A, David R, et al: Long-term ocular hypotensive effect of levobunolol: results of a one-year study. *Br J Ophthalmol* 1985;69:593-599.

161. Berson FG, Cohen HB, Foerster RJ, Lass JH, Novack GD, Duzman E: Levobunolol compared with timolol for the long-term control of elevated intraocular pressure. *Arch Ophthalmol* 1985;103:379-382.

162. Cinotti A, Cinotti D, Grant W, et al: Levobunolol vs timolol for open-angle glaucoma and ocular hypertension. *Am J Ophthalmol* 1985;99:11-17.

163. Dausch D, Gorlich W, Honegger H: [Clinical suitability of pindolol eyedrops in the treatment of chronic open-angle glaucoma]. *Klin Monatsbl Augenheilkd* 1984;184:539-542.

164. Merte HJ, Stryz JR, Mertz M: [Pindolol eye drops (Glauco-Visken) - half year's results in glaucoma therapy]. *Klin Monatsbl Augenheilkd* 1984;184:227-232.

165. Berry DPJ, van Buskirk EM, Shields MB: Betaxolol and timolol. A comparison of efficacy and side effects. *Arch Ophthalmol* 1984;102:42-45.

166. Duzman E, Rosen N, Lazar M: Diacetyl nadolol: 3-month ocular hypotensive effect in glaucomatous eyes. *Br J Ophthalmol* 1983;67:668-673.

167. Andreasson S, Jensen KM: Effect of pindolol on intraocular pressure in glaucoma: pilot study and a randomised comparison with timolol. *Br J Ophthalmol* 1983;67:228-230.

168. Mills KB: Blind randomised non-crossover long-term trial comparing topical timolol 0.25% with timolol 0.5% in the treatment of simple chronic glaucoma. *Br J Ophthalmol* 1983;67:216-219.

169. Duzman E, Ober M, Scharrer A, Leopold IH: A clinical evaluation of the effects of topically applied levobunolol and timolol on increased intraocular pressure. *Am J Ophthalmol* 1982;94:318-327.

170. Nielsen NV, Eriksen JS: Timolol and metoprolol in glaucoma. A comparison of the ocular hypotensive effect, local and systemic tolerance. *Acta Ophthalmol (Copenh)* 1981;59:336-346.

171. Merkle W: [Comparative study between timolol and d-epinephrine in the treatment of open-angle glaucoma (author's transl)]. *Klin Monatsbl Augenheilkd* 1981;178:203-205.

172. Thomas JV, Epstein DL: Timolol and epinephrine in primary open angle glaucoma. Transient additive effect. *Arch Ophthalmol* 1981;99:91-95.

173. Moss AP, Ritch R, Hargett NA, Kohn AN, Smith HJ, Podos SM: A comparison of the effects of timolol and epinephrine on intraocular pressure. *Am J Ophthalmol* 1978;86:489-495.

174. Bischoff P: [Experiences with timolol in treatment of glaucoma (author's transl)]. *Klin Monatsbl Augenheilkd* 1978;173:202-207.

### I d) Latanoprost

175. Chiba T, Kashiwagi K, Kogure S, et al: Iridial pigmentation induced by latanoprost ophthalmic solution in Japanese glaucoma patients. *J Glaucoma* 2001;10:406-410.

176. Saito M, Takano R, Shirato S: Effects of latanoprost and unoprostone when used alone or in combination for open-angle glaucoma. *Am J Ophthalmol* 2001;132:485-489.

177. Netland PA, Landry T, Sullivan EK, et al: Travoprost compared with latanoprost and timolol in patients with open-angle glaucoma or ocular hypertension. *Am J Ophthalmol* 2001;132:472-484.

178. Gandolfi S, Simmons ST, Sturm R, Chen K, VanDenburgh AM: Three-month comparison of bimatoprost and latanoprost in patients with glaucoma and ocular hypertension. *Adv Ther* 2001;18:110-121.

179. DuBiner H, Cooke D, Dirks M, Stewart WC, VanDenburgh AM, Felix C: Efficacy and safety of bimatoprost in patients with elevated intraocular pressure: a 30-day comparison with latanoprost. *Surv Ophthalmol* 2001;45 Suppl 4:S353-S360

180. Bron AM, Denis P, Nordmann JP, Rouland JF, Sellem E, Johansson M: Additive IOP-reducing effect of latanoprost in patients insufficiently controlled on timolol. *Acta Ophthalmol Scand* 2001;79:289-293.

181. Aung T, Chew PT, Yip CC, et al: A randomized double-masked crossover study comparing latanoprost 0.005% with unoprostone 0.12% in patients with primary open-angle glaucoma and ocular hypertension. *Am J Ophthalmol* 2001;131:636-642.

182. Stewart WC, Day DG, Stewart JA, Schuhr J, Latham KE: The efficacy and safety of latanoprost 0.005% once daily versus brimonidine 0.2% twice daily in open-angle glaucoma or ocular hypertension. *Am J Ophthalmol* 2001;131:631-635.

183. Konstas AG, Lake S, Maltezos AC, Holmes KT, Stewart WC: Twenty-four hour intraocular pressure reduction with latanoprost compared with pilocarpine as third-line therapy in exfoliation glaucoma. *Eye* 2001;15:59-62.

184. Polo V, Larrosa JM, Gomez ML, Pablo L, Honrubia FM: Latanoprost versus combined therapy with timolol plus dorzolamide: IOP-lowering effect in open-angle glaucoma. *Acta Ophthalmol Scand* 2001;79:6-9.

185. Susanna RJ, Giampani JJ, Borges AS, Vessani RM, Jordao ML: A double-masked, randomized clinical trial comparing latanoprost with unoprostone in patients with open-angle glaucoma or ocular hypertension.  *Ophthalmology* 2001;108:259-263.

186. Stewart WC, Day DG, Stewart JA, et al: Therapeutic success of latanoprost 0.005% compared to brimonidine 0.2% in patients with open-angle glaucoma or ocular hypertension. *J Ocul Pharmacol Ther* 2000;16:557-564.

187. Orzalesi N, Rossetti L, Invernizzi T, Bottoli A, Autelitano A: Effect of timolol, latanoprost, and dorzolamide on circadian IOP in glaucoma or ocular hypertension. *Invest Ophthalmol Vis Sci* 2000;41:2566-2573.

188. Hedman K, Alm A: A pooled-data analysis of three randomized, double-masked, six-month clinical studies comparing the intraocular pressure reducing effect of latanoprost and timolol. *Eur J Ophthalmol* 2000;10:95-104.

189. Aung T, Wong HT, Yip CC, Leong JY, Chan YH, Chew PT: Comparison of the intraocular pressure-lowering effect of latanoprost and timolol in patients with chronic angle closure glaucoma: a preliminary study. *Ophthalmology* 2000;107:1178-1183.

190. O'Donoghue EP: A comparison of latanoprost and dorzolamide in patients with glaucoma and ocular hypertension: a 3 month, randomised study. Ireland Latanoprost Study Group. *Br J Ophthalmol* 2000;84:579-582.

191. Alm A, Widengard I: Latanoprost: experience of 2-year treatment in Scandinavia. *Acta Ophthalmol Scand* 2000;78:71-76.

192. Thygesen J, Aaen K, Theodorsen F, Kessing SV, Prause JU: Short-term effect of latanoprost and timolol eye drops on tear fluid and the ocular surface in patients with primary open-angle glaucoma and ocular hypertension. *Acta Ophthalmol Scand* 2000;78:37-44.

193. Konstas AG, Maltezos AC, Gandi S, Hudgins AC, Stewart WC: Comparison of 24-hour intraocular pressure reduction with two dosing regimens of latanoprost and timolol maleate in patients with primary open-angle glaucoma. *Am J Ophthalmol* 1999;128:15-20.

194. Mastropasqua L, Carpineto P, Ciancaglini M, Gallenga PE: A 12-month, randomized, double-masked study comparing latanoprost with timolol in pigmentary glaucoma. *Ophthalmology* 1999;106:550-555.

195. Camras CB, Wax MB, Ritch R, et al: Latanoprost treatment for glaucoma: effects of treating for 1 year and of switching from timolol. United States Latanoprost Study Group. *Am J Ophthalmol* 1998;126:390-399.

196. Widengard I, Maepea O, Alm A: Effects of latanoprost and dipivefrin, alone or combined, on intraocular pressure and on blood-aqueous barrier permeability. *Br J Ophthalmol* 1998;82:404-406.

197. Fristrom B, Nilsson SE: A double masked comparison of the intraocular pressure reducing effect of latanoprost 0.005% and 0.001% administered once daily in open angle glaucoma and ocular hypertension. *Br J Ophthalmol* 1997;81:867-870.

198. Watson PG: Latanoprost. Two years' experience of its use in the United Kingdom. Latanoprost Study Group. *Ophthalmology* 1998;105:82-87.

199. Lusky M, Ticho U, Glovinsky J, et al: A comparative study of two dose regimens of latanoprost in patients with elevated intraocular pressure. *Ophthalmology* 1997;104:1720-1724.

200. Diestelhorst M, Roters S, Krieglstein GK: The effect of latanoprost 0.005% once daily versus 0.0015% twice daily on intraocular pressure and aqueous humour protein concentration in glaucoma patients. A randomized, double-masked comparison with timolol 0.5%. *Graefes Arch Clin Exp Ophthalmol* 1997;235:20-26.

201. Camras CB, Alm A, Watson P, Stjernschantz J: Latanoprost, a prostaglandin analog, for glaucoma therapy. Efficacy and safety after 1 year of treatment in 198 patients. Latanoprost Study Groups. *Ophthalmology* 1996;103:1916-1924.

202. Mishima HK, Masuda K, Kitazawa Y, Azuma I, Araie M: A comparison of latanoprost and timolol in primary open-angle glaucoma and ocular hypertension. A 12-week study. *Arch Ophthalmol* 1996;114:929-932.

203. Fristrom B: A 6-month, randomized, double-masked comparison of latanoprost with timolol in patients with open angle glaucoma or ocular hypertension. *Acta Ophthalmol Scand* 1996;74:140-144.

204. Camras CB: Comparison of latanoprost and timolol in patients with ocular hypertension and glaucoma: a six-month masked, multicenter trial in the United States. The United States Latanoprost Study Group. *Ophthalmology* 1996;103:138-147.

205. Watson P, Stjernschantz J: A six-month, randomized, double-masked study comparing latanoprost with timolol in open-angle glaucoma and ocular hypertension. The Latanoprost Study Group. *Ophthalmology* 1996;103:126-137.

206. Alm A, Stjernschantz J: Effects on intraocular pressure and side effects of 0.005% latanoprost applied once daily, evening or morning. A comparison with timolol. Scandinavian Latanoprost Study Group. *Ophthalmology* 1995;102:1743-1752.

## II) Raw data of the studies included in the meta-Analysis

### II a) Pravastatin

| **Nr. of the publication in index I a)** | **Year of publication** | **Reported effect size (lowering of LDL-C in percent)** | **Baseline**  **(LDL-C in mg/dl)** | **Study size (number of patients)** | **Treatment group (experimental or control group)** |
| --- | --- | --- | --- | --- | --- |
| 1 | 2001 | 26 | 200.3 | 37 | V |
| 2 | 2001 | 21 | 170 | 22 | V |
| 3 | 2001 | 31.5 | 183.3 | 1170 | V |
| 4 | 2001 | 19 | 179.8 | 31 | E |
| 5 | 2001 | 23 | 134.4 | 1702 | E |
| 6 | 2001 | 21.7 | 169.7 | 22 | V |
| 7 | 2000 | 25.24 | 150.4 | 3260 | E |
| 8 | 2000 | 30.3 | 181.2 | 1030 | V |
| 9 | 2000 | 25 | 157 | 25 | E |
| 10 | 2000 | 29.3 | 155.8 | 45 | E |
| 11 | 2000 | 30.1 | 182 | 159 | E |
| 12 | 1999 | 26.3 | 205.3 | 387 | E |
| 13 | 1999 | 33 | 160.5 | 60 | E |
| 14 | 1999 | 25 | 150 | 9014 | E |
| 15 | 1998 | 27.5 | 136 | 586 | E |
| 16 | 1998 | 28 | 144.5 | 1283 | E |
| 17 | 1998 | 29.5 | 183.7 | 19 | E |
| 18 | 1998 | 28 | 144.5 | 576 | E |
| 19 | 1998 | 27 | 220 | 522 | E |
| 20 | 1998 | 32 | 212.7 | 2036 | E |
| 21 | 1998 | 34 | 215 | 534 | V |
| 22 | 1997 | 20.2 | 163 | 13 | E |
| 23 | 1996 | 22 | 180 | 305 | E |
| 24 | 1996 | 32 | 139 | 4159 | E |
| 25 | 1996 | 30 | 235.9 | 20 | E |
| 26 | 1996 | 34 | 212.7 | 147 | E |
| 27 | 1995 | 26 | 192 | 6595 | E |
| 28 | 1995 | 28 | 164 | 408 | E |
| 29 | 1995 | 30.6 | 199 | 32 | E |
| 30 | 1995 | 27.4 | 189.5 | 424 | E |
| 31 | 1995 | 26.6 | 226 | 134 | V |
| 32 | 1995 | 31 | 212 | 20 | V |
| 33 | 1995 | 30 | 216.5 | 22 | E |
| 34 | 1995 | 34.2 | 171.7 | 38 | E |
| 35 | 1995 | 28 | 166 | 241 | E |
| 36 | 1995 | 35 | 216.5 | 19 | E |
| 37 | 1994 | 25 | 241.7 | 30 | E |
| 38 | 1994 | 31.1 | 214.7 | 48 | E |
| 39 | 1994 | 32.1 | 210.7 | 33 | E |
| 40 | 1994 | 29.9 | 201.7 | 22 | E |
| 41 | 1994 | 30 | 197 | 50 | E |
| 42 | 1994 | 33 | 227 | 158 | E |
| 43 | 1993 | 30 | 170.1 | 32 | E |
| 44 | 1993 | 33 | 314 | 48 | E |
| 45 | 1993 | 31.3 | 235.9 | 311 | E |
| 46 | 1993 | 27 | 196 | 672 | E |
| 47 | 1993 | 27.2 | 234.3 | 51 | E |
| 48 | 1993 | 36.8 | 197.6 | 24 | E |
| 49 | 1993 | 33.5 | 200.7 | 290 | E |
| 50 | 1992 | 30 | 319.8 | 60 | E |
| 51 | 1992 | 24.4 | 344 | 19 | E |
| 52 | 1992 | 30 | 302.4 | 128 | E |
| 53 | 1992 | 31.3 | 237.3 | 29 | E |
| 54 | 1991 | 31 | 200 | 82 | E |
| 55 | 1991 | 37 | 216 | 26 | E |
| 56 | 1991 | 39 | 180.6 | 10 | E |
| 57 | 1991 | 34.1 | 208.4 | 150 | E |
| 58 | 1991 | 30 | 278.4 | 385 | E |
| 59 | 1990 | 33.2 | 302 | 55 | E |
| 60 | 1990 | 31.9 | 245.6 | 196 | E |
| 61 | 1990 | 32.1 | 227 | 40 | E |
| 62 | 1990 | 33 | 336.4 | 40 | E |
| 63 | 1990 | 35 | 208.9 | 33 | E |
| 64 | 1990 | 29 | 191.8 | 10 | E |

### II b) Atorvastatin

| **Nr. of the publication in index I b)** | **Year of publication** | **Reported effect size (lowering of LDL-C in percent)** | **Baseline**  **(LDL-C in mg/dl)** | **Study size (number of patients)** | **Treatment group (experimental or control group)** |
| --- | --- | --- | --- | --- | --- |
| 65 | 2001 | 41.6 | 192.7 | 54 | E |
| 66 | 2001 | 32 | 170 | 22 | E |
| 67 | 2001 | 33.9 | 169.8 | 29 | E |
| 68 | 2001 | 38 | 182 | 94 | V |
| 69 | 2001 | 37.7 | 203.2 | 215 | V |
| 70 | 2001 | 36 | 179 | 3916 | E |
| 71 | 2001 | 44.2 | 189.4 | 142 | V |
| 72 | 2001 | 37.15 | 187.9 | 328 | E |
| 73 | 2001 | 40.8 | 143 | 217 | E |
| 74 | 2001 | 28.4 | 195.5 | 96 | E |
| 75 | 2001 | 34.8 | 224.3 | 235 | E |
| 76 | 2001 | 35 | 189.1 | 42 | E |
| 77 | 2001 | 32.9 | 169.7 | 22 | E |
| 78 | 2001 | 37.2 | 181.2 | 1424 | E |
| 79 | 2001 | 37 | 213 | 409 | E |
| 80 | 2001 | 42 | 185 | 30 | V |
| 81 | 2001 | 29 | 265.3 | 53 | E |
| 82 | 2000 | 36.7 | 193.4 | 258 | E |
| 83 | 2000 | 35 | 145 | 22 | E |
| 84 | 2000 | 37 | 247 | 272 | E |
| 85 | 1999 | 37.9 | 233.8 | 197 | E |
| 86 | 1999 | 35.3 | 198 | 91 | E |
| 87 | 1998 | 36 | 205 | 344 | E |
| 88 | 1998 | 33 | 173 | 318 | E |
| 89 | 1998 | 33 | 340.3 | 136 | E |
| 90 | 1998 | 30 | 171 | 108 | E |
| 91 | 1998 | 38 | 213 | 534 | E |
| 92 | 1997 | 30 | 187.2 | 83 | E |
| 93 | 1997 | 41 | 190.5 | 55 | E |
| 94 | 1997 | 37 | 211.5 | 177 | E |
| 95 | 1997 | 36 | 192.2 | 1049 | E |
| 96 | 1997 | 38 | 221 | 108 | E |
| 97 | 1997 | 35 | 195 | 305 | E |
| 98 | 1996 | 35 | 187 | 39 | E |
| 99 | 1996 | 41 | 187.9 | 81 | E |

### II c) Timolol

| **Nr. of the publication in index I c)** | **Year of publication** | **Reported effect size (lowering of IOP in mmHg)** | **Baseline**  **(IOP in mmHg)** | **Study size (number of patients)** | **Treatment group (experimental or control group)** |
| --- | --- | --- | --- | --- | --- |
| 100 | 2001 | 5.9 | 25.7 | 801 | V |
| 101 | 2001 | 7.1 | 26.1 | 573 | V |
| 102 | 2001 | 3.65 | 25.1 | 100 | V |
| 103 | 2001 | 6.74 | 25.7 | 596 | V |
| 104 | 2001 | 5.6 | 24.3 | 1198 | V |
| 105 | 2001 | 6.45 | 26.7 | 286 | V |
| 106 | 2001 | 4 | 22.1 | 12 | E |
| 107 | 2000 | 4.5 | 20.8 | 40 | V |
| 108 | 2000 | 6.7 | 27.1 | 90 | E |
| 109 | 2000 | 4 | 22.7 | 20 | V |
| 110 | 2000 | 6.2 | 25.4 | 219 | V |
| 111 | 2000 | 5.7 | 25.15 | 32 | V |
| 112 | 2000 | 6.5 | 25 | 829 | V |
| 113 | 2000 | 5.15 | 25.4 | 378 | V |
| 114 | 2000 | 6.17 | 24 | 45 | V |
| 115 | 2000 | 5.57 | 25.5 | 94 | V |
| 116 | 1999 | 4 | 23.1 | 34 | V |
| 117 | 1999 | 4.8 | 24.1 | 36 | V |
| 118 | 1999 | 6.2 | 25 | 837 | V |
| 119 | 1999 | 6 | 25.6 | 202 | V |
| 120 | 1998 | 6.9 | 24.4 | 36 | V |
| 121 | 1998 | 6.4 | 28.8 | 335 | V |
| 122 | 1998 | 5.9 | 25.2 | 483 | V |
| 123 | 1998 | 5 | 24.1 | 248 | V |
| 124 | 1998 | 5.75 | 25.9 | 572 | V |
| 125 | 1998 | 8 | 25 | 277 | V |
| 126 | 1997 | 5.6 | 25.2 | 176 | V |
| 127 | 1997 | 6.1 | 24.6 | 374 | V |
| 128 | 1997 | 5.4 | 23.7 | 154 | V |
| 129 | 1997 | 4.7 | 29.2 | 28 | V |
| 130 | 1996 | 6 | 24.64 | 926 | V |
| 131 | 1996 | 4.4 | 22.1 | 184 | V |
| 132 | 1996 | 5.6 | 24.55 | 171 | V |
| 133 | 1996 | 6.8 | 25 | 371 | V |
| 134 | 1996 | 8.9 | 27.05 | 209 | V |
| 135 | 1996 | 6.6 | 24.8 | 31 | V |
| 136 | 1996 | 11.3 | 38.7 | 16 | V |
| 137 | 1996 | 8.3 | 25.4 | 294 | V |
| 138 | 1996 | 4.9 | 25.3 | 258 | V |
| 139 | 1995 | 6.7 | 24.6 | 267 | V |
| 140 | 1995 | 5.5 | 22 | 523 | V |
| 141 | 1995 | 6.2 | 24.8 | 25 | V |
| 142 | 1993 | 5 | 26.1 | 69 | V |
| 143 | 1993 | 5.4 | 23.7 | 158 | V |
| 144 | 1992 | 7.9 | 23.7 | 72 | V |
| 145 | 1991 | 6.5 | 24.8 | 105 | V |
| 146 | 1989 | 7 | 27.1 | 391 | V |
| 147 | 1988 | 8.8 | 28 | 51 | V |
| 148 | 1988 | 6 | 25.11 | 26 | V |
| 149 | 1988 | 6.7 | 27.4 | 27 | V |
| 150 | 1988 | 7.2 | 29.5 | 41 | V |
| 151 | 1988 | 7.9 | 28 | 47 | E |
| 152 | 1988 | 5.2 | 26.7 | 40 | V |
| 153 | 1987 | 7.1 | 24.2 | 28 | V |
| 154 | 1986 | 9.3 | 28.7 | 51 | V |
| 155 | 1986 | 4.3 | 25.3 | 18 | V |
| 156 | 1986 | 8.4 | 29 | 29 | V |
| 157 | 1985 | 5.05 | 23.95 | 57 | E |
| 158 | 1985 | 5.1 | 28.1 | 50 | V |
| 159 | 1985 | 7.1 | 27.1 | 391 | V |
| 160 | 1985 | 6 | 26.4 | 88 | V |
| 161 | 1985 | 7.6 | 27 | 141 | V |
| 162 | 1985 | 8 | 27.3 | 162 | V |
| 163 | 1984 | 7.8 | 25.5 | 42 | E |
| 164 | 1984 | 6.4 | 24.3 | 81 | E |
| 165 | 1984 | 9.9 | 29.8 | 46 | V |
| 166 | 1983 | 8.6 | 27.6 | 31 | V |
| 167 | 1983 | 8 | 25 | 28 | E |
| 168 | 1983 | 5.4 | 24.8 | 30 | E |
| 169 | 1982 | 8.9 | 28.6 | 16 | V |
| 170 | 1981 | 8.7 | 28 | 19 | V |
| 171 | 1981 | 8.7 | 27.2 | 40 | E |
| 172 | 1981 | 9 | 31 | 16 | E |
| 173 | 1978 | 7.7 | 28.3 | 36 | E |
| 174 | 1978 | 8.6 | 28.7 | 40 | E |

### I d) Latanoprost

| **Nr. of the publication in index I d)** | **Year of publication** | **Reported effect size (lowering of IOP in mmHg)** | **Baseline**  **(IOP in mmHg)** | **Study size (number of patients)** | **Treatment group (experimental or control group)** |
| --- | --- | --- | --- | --- | --- |
| 175 | 2001 | 5.4 | 20 | 69 | E |
| 176 | 2001 | 6 | 22.9 | 52 | E |
| 177 | 2001 | 7 | 25.7 | 801 | V |
| 178 | 2001 | 7.6 | 25.7 | 232 | V |
| 179 | 2001 | 5.48 | 22.9 | 64 | V |
| 180 | 2001 | 6.7 | 26.3 | 53 | E |
| 181 | 2001 | 6.1 | 22.3 | 56 | E |
| 182 | 2001 | 5.7 | 21.5 | 33 | E |
| 183 | 2001 | 3.5 | 21.5 | 30 | E |
| 184 | 2001 | 5.29 | 22.06 | 35 | E |
| 185 | 2001 | 6.7 | 24.1 | 108 | E |
| 186 | 2000 | 4.5 | 21.6 | 157 | E |
| 187 | 2000 | 6.4 | 22.7 | 20 | E |
| 188 | 2000 | 7.7 | 24.8 | 829 | E |
| 189 | 2000 | 8.8 | 25.7 | 30 | E |
| 190 | 2000 | 8.5 | 27.2 | 213 | E |
| 191 | 2000 | 7.7 | 25.1 | 183 | E |
| 192 | 2000 | 5.7 | 21.6 | 37 | E |
| 193 | 1999 | 5.2 | 23.1 | 34 | E |
| 194 | 1999 | 5.9 | 24.8 | 36 | E |
| 195 | 1998 | 7 | 24.4 | 223 | E |
| 196 | 1998 | 4.5 | 19.3 | 22 | E |
| 197 | 1998 | 9.6 | 27.4 | 24 | E |
| 198 | 1998 | 8 | 25.2 | 227 | E |
| 199 | 1997 | 7.5 | 24.7 | 50 | E |
| 200 | 1997 | 9.8 | 28.2 | 30 | E |
| 201 | 1996 | 7.9 | 25.3 | 198 | E |
| 202 | 1996 | 6.2 | 23.1 | 184 | E |
| 203 | 1996 | 8.75 | 25.9 | 31 | E |
| 204 | 1996 | 6.7 | 24.8 | 268 | E |
| 205 | 1996 | 8.5 | 25.2 | 294 | E |
| 206 | 1995 | 8.2 | 25.15 | 267 | E |
